# MTIE-Net: Multi-technology fusion of low-light image enhancement network

**DOI:** 10.1371/journal.pone.0297984

**Published:** 2024-02-02

**Authors:** Jing Tao, Hao Wu, Zhihao Ni, Zhongyang Jin, Changhua Zhong

**Affiliations:** 1 Automation and Information School of Automation and Information Engineering, Sichuan University of Science & Engineering, Zigong, Sichuan Province, China; 2 Artificial Intelligence Key Laboratory of Sichuan Province, Zigong, Sichuan Province, China; Wuhan University of Science and Technology, CHINA

## Abstract

Images obtained in low-light scenes are often accompanied by problems such as low visibility, blurred details, and color distortion, enhancing them can effectively improve the visual effect and provide favorable conditions for advanced visual tasks. In this study, we propose a Multi-Technology Fusion of Low-light Image Enhancement Network (MTIE-Net) that modularizes the enhancement task. MTIE-Net consists of a residual dense decomposition network (RDD-Net) based on Retinex theory, an encoder-decoder denoising network (EDD-Net), and a parallel mixed attention-based self-calibrated illumination enhancement network (PCE-Net). The low-light image is first decomposed by RDD-Net into a lighting map and reflectance map; EDD-Net is used to process noise in the reflectance map; Finally, the lighting map is fused with the denoised reflectance map as an input to PCE-Net, using the Fourier transform for illumination enhancement and detail recovery in the frequency domain. Numerous experimental results show that MTIE-Net outperforms the comparison methods in terms of image visual quality enhancement improvement, denoising, and detail recovery. The application in nighttime face detection also fully demonstrates its promise as a pre-processing means in practical applications.

## 1. Introduction

With the rapid development of deep learning, digital image processing technology has been widely used in industrial production, video surveillance, daily life, military applications and other fields. However, there are frequently objective factors that lead to image defects during the image acquisition process. For instance, under weak illumination conditions, such as at night or on cloudy days, the light reflected from the surface of objects is generally weak. This often results in images that are characterized by low brightness, low contrast, color distortion, and high noise, as demonstrated in studies [1–3]. In addition, for color low-light images, the pixel values are mainly concentrated in a low range, and the gray scale difference of the corresponding pixels between each channel is also very limited, with only a small difference between the maximum and minimum gray scale of the image, a deviation of the overall color layer, and weak edge information, which leads to the difficulty in distinguishing the details of the image when observed by human beings or processed by computers. These characteristics seriously reduce the subjective visual effects of low-light images, resulting in a significant reduction in their usability [4]. Enhancing low-light images and converting them into high-quality clear images can effectively improve the performance of advanced visual tasks such as target detection, multi-target tracking, semantic segmentation, underwater vision [5, 6] and face recognition. There-fore, low-light image enhancement technology has important research significance and broad application prospects as a pre-processing to improve subsequent advanced vision tasks. The purpose of low-light image enhancement is to improve its visibility, transforming the image into a form more suitable for human observation or computer pro-cessing, while suppressing noise and artifacts. At present, in order to weaken the influence of low-light images on visual tasks, researchers have proposed two solutions. One is to improve the performance of image acquisition equipment [7, 8], and the other is to perform illumination enhancement processing on the collected low-light images. Existing low-light cameras from companies such as Sony, Photonis, SiOnyx, and Texas Instruments use high-performance charge-coupled devices or complementary met-al-oxide-semiconductor [9] technology, professional low-light circuits, and filters as core components to improve low-light image quality. However, due to the demanding manufacturing process, complex technology and high price of these high-performance equipment, they have not been widely used at this stage [10]. As an alternative, the use of algorithms to perform illumination enhancement on low-illumination images provides greater flexibility [11].

Over the past few decades, there has been significant progress in the research of low-light image enhancement. However, these methods are not stable in unknown application scenarios, and there is still a large room for improvement in general. For example, the noise amplification caused during the enhancement process leads to blurred details and color deviations in the enhancement results; when improving image contrast, the appropriate balance between image color, visual effects, information entropy and other factors is not considered. In summary, we propose a Multi-Technology Fusion of Low-light Image Enhancement Network (MTIE-Net). The network modularizes the enhancement task, i.e., decomposition, denoising and enhancement. Among them, the decomposition network combines dense residual blocks for global residual learning; The denoising network adopts an encoder-decoder structure, and uses channel Transformer instead of simple skip connections to transfer feature information to improve the denoising performance of the network. The enhancement network exploits the frequency information of the image. A new attentional mechanism is used for weighted fusion of different regions in an image for the purpose of enhancement. Furthermore, the enhancement process incorporates a self-calibrated training strategy. This strategy continuously adjusts the inputs at each stage, ensuring rapid convergence to an optimal level.

Overall, the contributions of this paper are as follows.

1. We propose a Multi-Technology Fusion of Low-light Image Enhancement Network (MTIE-Net). MTIE-Net contains a dense residual decomposition network based on Retinex theory (RDD-Net), a denoising network based on encoder-decoder structure (EDD-Net) and a self-calibrated enhancement network based on parallel mixed attention mechanism (PCE-Net). Among them, RDD-Net decomposes the input low-light image into lighting map and reflectance map; EDD-Net is used to process the noise in the reflectance map; finally, the lighting map is fused with the denoised reflectance map as the input of PCE-Net, and the Fourier Transform is used to perform the illumination enhancement and detail recovery in the frequency domain.
2. We study a residual dense block to construct the more efficient residual dense decomposition network by utilizing the powerful feature extraction capability of residual dense network to facilitate the feature information transfer between channels. In the design of EDD-Net, we use subpixel convolution for upsampling to improve the resolution of the image while denoising; The channel Transformer is used instead of skip connections in the traditional encoder-decoder architecture to better capture remote dependencies and enhance contextual understanding. In addition, we propose a new attentional mechanism that enables PCE-Net to adaptively perform weighted fusion of different regions in an image to improve the enhancement effect.
3. Numerous experimental results show that MTIE-Net outperforms the comparison methods in terms of image visual quality enhancement improvement, denoising and detail recovery. The application of MTIE-Net as a pre-processing method to face detection at night further validates its application value in improving the performance of advanced visual tasks.

### 1.1 Related work

Existing research on low-light image enhancement categorizes methods into two primary types: traditional methods and network-based methods. Traditional methods are mainly based on histogram equalization and the Retinex theory. The use of histogram equalization to stretch the dynamic range of an image was first proposed by Pizer et al. [12] for the enhancement of low-light images. The method is computationally simple but cannot suppress noise and has limited effect on low-light image enhancement. Jobson et al. [13] were the first to apply the Retinex [14] theory to the field of low-light image enhancement, proposing to decompose the image into lighting map and reflectance map, and then perform the enhancement process. The method achieves favorable results in noise suppression and detail recovery, but due to the large computational volume of the algorithm, it is unable to process batch low-light images quickly. Later, Chen et al. [15] combined CNN with Retinex theory and proposed RetinexNet. The network contains two modules, decomposition and illumination enhancement. The decomposition module decomposes the low-light image into lighting map and reflectance map; the illumination enhancement module is responsible for enhancing the low-light image and denoising the reflectance map before outputting the reconstructed image. In recent years, deep neural networks have been widely used in the field of image enhancement with good results due to their powerful nonlinear fitting ability [16]. Jiang Hai et al. [17] proposed a novel Real-low to Real-normal Network (R2RNet) based on Retinex theory. Combining the spatial and frequency information of the image for illumination enhancement and proposing a new frequency loss function to recover more image details. Guo et al. [18] proposed Zero Reference Depth Curve Estimation (Zero-DCE), which uses low-light image enhancement as a deep network-based image-specific curve estimation task. The method takes a low-light image as input and produces a higher order curve as its output, then adjusts the dynamic range of the original image pixel by pixel based on the output curve. It achieves low-light image enhancement through intuitive and simple nonlinear curve mapping. Xu et al. [19] designed a signal-to-noise ratio (SNR) aware converter with a new self-attention module and combined it with a convolutional model through spatial variations to achieve dynamic enhancement of low-light images with a SNR prior. Wang et al. [20] proposed a flow-based low-light image enhancement method (LLFlow). By modeling the distribution of normally exposed images as an invertible network of Gaussian distributions and using this to learn a one-to-many mapping from low-light images to the distribution of normally exposed images. The method enables better regulation of light as well as suppression of noise and artifacts. Ma et al. [21] proposed a new Self-Calibrating Illumination (SCI) learning framework, which employs a cascading illumination learning process with weight sharing to deal with low-light image enhancement tasks. Among them, the self-calibration module can greatly reduce the computational burden of the cascade mode and realize fast, flexible and stable brightening of images in real low-light scenes. Zhang et al. [22] used a dual-histogram-based iterative threshold method and a limited histogram method with Rayleigh distribution to improve the global and local contrast of the color-corrected image. After multi-scale fusion of the two, a multiscale unsharp masking strategy to further sharpen the fused image for better visual quality.

## 2 Methods

### 2.1 Network architecture

**Fig 1:**
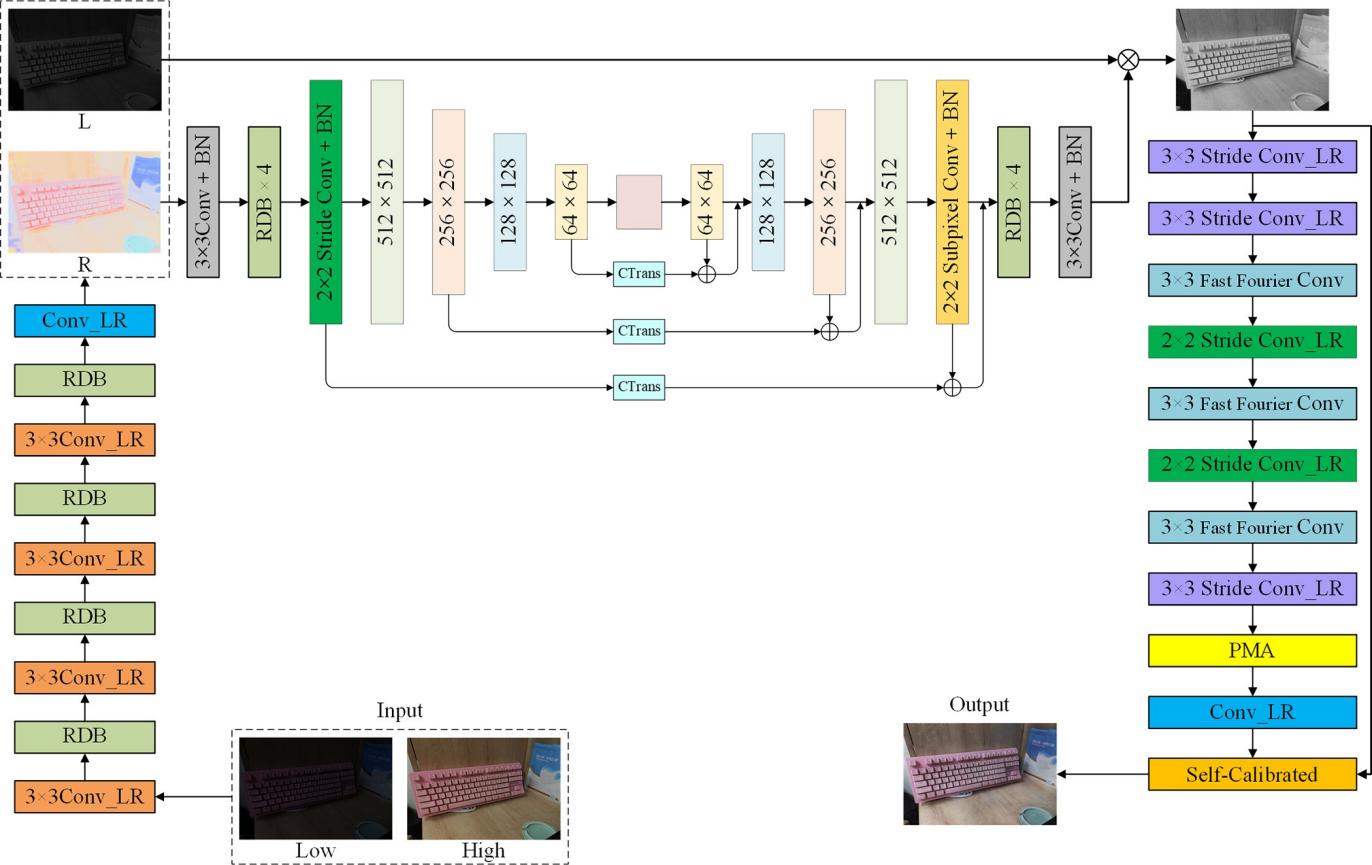
The network structure of the improved MTIE-Net.

Drawing inspiration from the works of [15, 17], MTIE-Net comprises three main stages: decomposition, denoising, and enhancement. As illustrated in Fig 1: the input low-light image is firstly decomposed into lighting map and reflectance map, so as to realize the effective separation of image features and noise. Subsequently, the reflectance map is denoised, and then the denoised reflectance map is multiplied element-by-element with the lighting map, and the result obtained is used as the input to the enhancement network, which utilizes the Fourier transform to perform illumination enhancement and detail recovery in the frequency domain.

#### 2.1.1 RDD-Net

The Retinex theory [23] proposes a basic physical law for low-light image enhancement, i.e., the visual color image *I* can be decomposed into reflectance map *R* and lighting map *L*, as shown in Eq (1):

I=R⊗L (1)

Where ⊗ denotes element-by-element multiplication. The reflectance map is an inherent property of the image and is consistent across all luminance conditions; The lighting map represents the various luminances on an object, which are usually affected by darkness and unbalanced illumination distributions. Therefore, illumination enhancement can be achieved by changing the dynamic range of the pixels in the lighting map [15].

Retinex-based methods have been shown to be effective means of low-light image enhancement. However, in several early Retinex-based methods [13, 14, 24], the lighting map is first estimated and then the reflectance map is used as the final enhancement result. Although it can significantly restore image details, often results in unnatural and overexposed visual effects [25, 26]. In addition, existing Retinex-based decomposition networks generally control the performance through elaborate constraints and parameters, resulting in reduced model robustness across various application scenarios [15].

**Fig 2:**
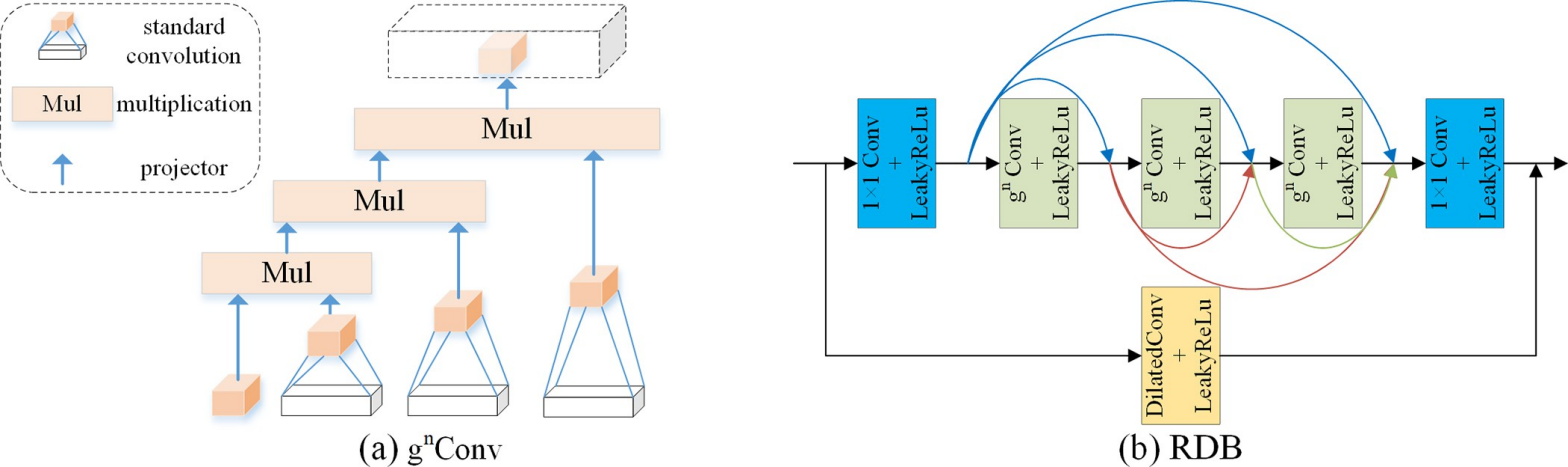
The network structure of RDB.

Studies [27, 28] have demonstrated that residual dense networks [29] and long skip connections can be used to construct deep convolutional neural networks to improve the performance of visual tasks. In order to obtain higher quality lighting maps and reflectance maps, this paper combines Residual Dense Block (RDB) with Retinex theory to construct a more efficient residual dense decomposition network (RDD-Net). Among them, the RDB consists of standard convolution with Recursive Gated Convolution (g^n^Conv) [30], using LeakyReLu as the activation function. The structure of the RDB network is illustrated in Fig 2(B): firstly, shallow features are extracted using a 1 × 1 convolution, and the output information is adaptively controlled for local feature fusion to further improve the information flow through local residual learning; Secondly, the shallow features are used as inputs, and 3 sequential g^n^Conv are used to extract the deep features, using their recursive design to improve the spatial interaction between the shallow features and the deep features; Afterwards, continuous feature transfer is generated through dense connectivity, which fully utilizes the features of all previous layers for dense feature fusion; Then a 1 × 1 convolution is used for global feature fusion; Finally the output feature map of the RDB is fused with the shallow features of the original input image through a dilation convolution (with a dilation factor of 2) via skip connection in order to maintain the structural similarity between the decomposed image and the original image [31]. Dilated convolution, in contrast to standard convolution, expands the sensory field without sacrificing resolution, enabling the extraction of more localized features.

#### 2.1.2 EDD-Net

Current deep learning based low-light image enhancement methods are learned based on datasets with minimal noise [32]. However, images captured in actual low-light conditions often contain varying levels of noise, which is the main reason why such methods are usually ineffective in unknown and complex scenes. To address this, we introduce a denoising network with an encoder-decoder structure, termed EDD-Net. As a plug-and-play sub-network, EDD-Net can rapidly and flexibly handle different levels of noise generated in the enhancement process of low-light images, so that the enhancement network can be interfered by as little noise as possible in the illumination recovery process.

**Fig 3:**
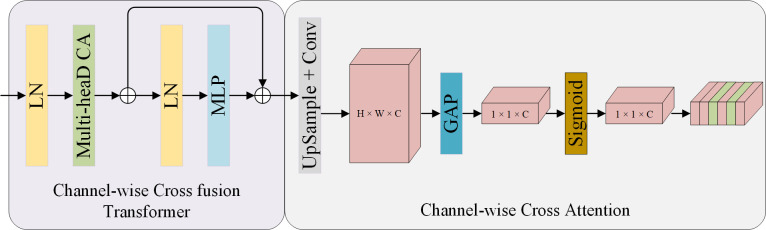
The network structure of CTrans.

The backbone of EDD-Net is based on the Res_U-Net [33] architecture, which takes advantage of the high efficiency of the encoder-decoder structure for transforming images to remove noise from reflectance maps. where the encoder structure captures both low-level and high-level features; The decoder structure combines semantic features to construct the final output; The Channel Transformer (CTrans) module [34] is used to convey the spatial information lost in the pooling layer and restore the complete spatial resolution through the encoding-decoding process. As illustrated in Fig 3, CTrans consists of a Channel-wise Cross fusion Transformer and a Channel-wise Cross Attention. The former is used for multi-scale fusion of features in the encoder structure; the latter is used for fusing features that are semantically inconsistent between the decoder and decoder structures. EDD-Net operates across four scales (512, 256, 128, and 64 channels), utilizing four consecutive RDBs for up/down sampling. Redundant low-frequency information is bypassed by additional skip connections between each up/down sampling, allowing the network to focus on learning high-frequency information. In this, the downsampling process converts the noisy image from a high resolution scale to a low resolution scale using step convolution; The upsampling process uses Subpixel Convolution to transfer the low resolution scale back to the high resolution scale, thus removing noise and preserving important features of the input image.

#### 2.1.3 PCE-Net

According to Retinex theory, the low-lighting image *i* is associated with the desired clear image *z* as follows:

i=z⊗l (2)

Where *l* denotes the lighting component. In general, enhancing the lighting component is regarded as the primary means of low-light image enhancement. Therefore, in this paper, we begin with the lighting map derived from RDD-Net decomposition, first fusing it with the denoised reflectance map from EDD-Net, and then in the illumination enhancement. Considering that existing low-light enhancement [18–20] focuses more on the spatial information of the image and ignores the channel information during the design, resulting in the enhanced image still having problems such as blurring of details, we study a self-calibrated illumination enhancement network based on parallel mixed attention (PCE-Net). Drawing inspiration from [17, 35], PCE-Net initially converts the input image from the spatial to the frequency domain using the Fourier transform to obtain the frequency component information of the image. Among them, the high amplitude frequency component implies that in the spatial domain image, there is a faster change in luminance or color, which usually corresponds to information such as details, edges, textures, etc. in the image; Low amplitude frequency domain components are used to recognize the global structural information of the image, such as the overall shape, general distribution in the image, etc. Then, Fast Fourier Convolution (FFC) [36] is used to integrate the magnitude and phase information of the input image in the frequency domain and to realize the detail reconstruction by enhancing the high magnitude frequency components. Finally, the Fourier inverse transform is utilized to convert the image back to the spatial domain and output the enhanced high frequency signal.

**Fig 4:**
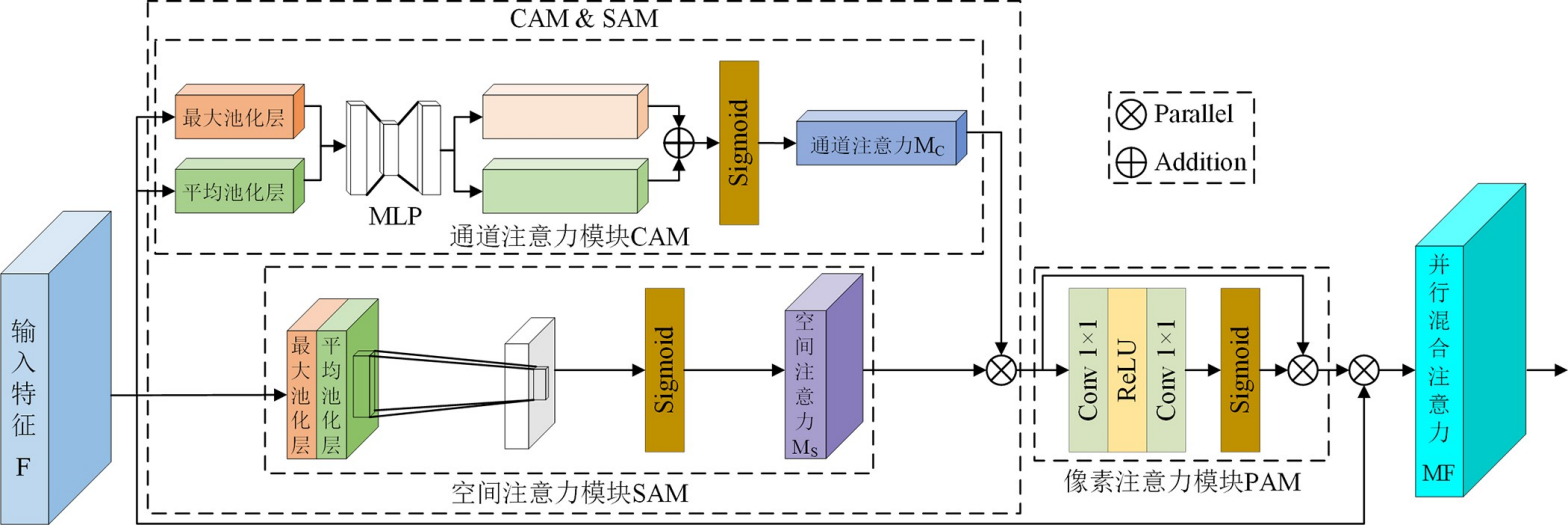
The network structure of PMA.

In order to take into account the channel information of the image during the enhancement process, we study a parallel mixed attention module (PMA). As illustrated in Fig 4, PMA consists of the Pixel Attention Module (PAM) [37] fused with the Channel Attention Module (CAM) and Spatial Attention Module (SAM) mechanisms of the parallel structure, which can simultaneously attend to the channel, spatial, and pixel information of the images. In Section 3.3.1, the tandem structure of CAM and SAM suffers from distribution lag in feature extraction, which leads to an overall distortion of the color of the enhanced low-light image, whereas connecting the two in parallel focuses on both the light information and the color information of the low-light image. In addition, it was shown in [16] that low-light images have extremely high local dependencies between neighboring pixel points, While PAM can adaptively rescale the per-pixel weights for all input feature mappings. For this reason, we integrated PAM behind the parallel structure of CAM and SAM to adjust all input features channel-by-channel and pixel-by-pixel. The characterization strength of pixel information in low-light images is improved while strengthening the feature linkage between adjacent pixel points, which in turn improves the overall performance of PCE-Net.

**Fig 5:**
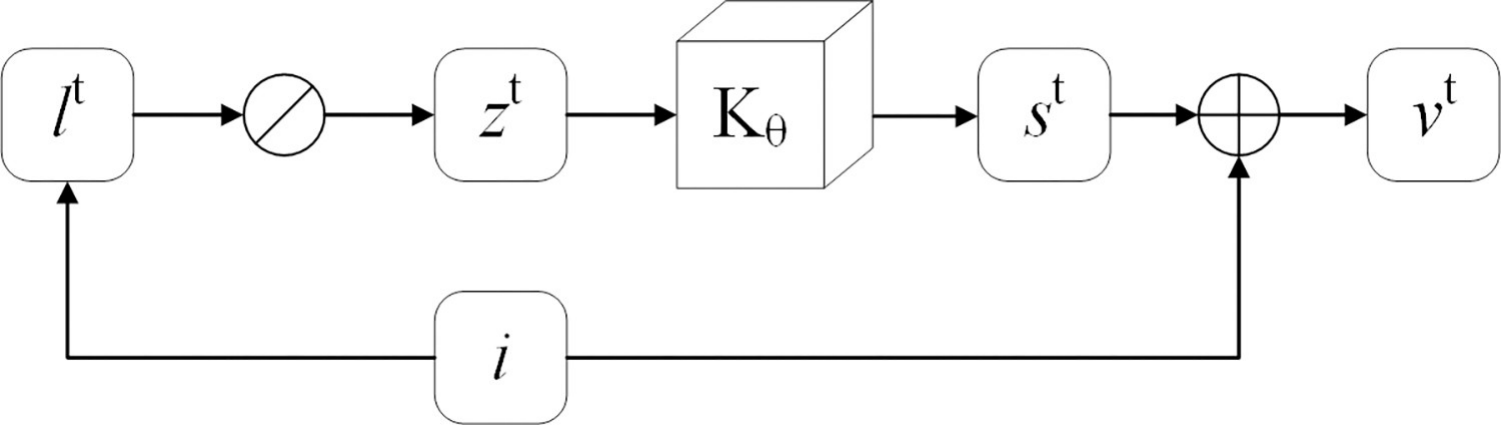
The calibration process.

Furthermore, PCE-Net employs a Self-Calibrated (SC) [21, 38] training strategy, which continuously calibrates the input image throughout the enhancement process to converge the results at each stage, which improves the exposure stability while greatly reducing the computational effort. Essentially, the core idea of SC is to introduce an auxiliary process in the training phase to enhance the modeling capabilities of the augmented network. This module extracts features from the input image (at the first stage) using multiple convolutions, serving as the input for the subsequent stage of illumination estimation and generating calibration results; Connecting the inputs of each subsequent stage to the inputs of the first stage ensures that the outputs of the different stages in the training process converge to the same state, and ultimately calibrates the input images to improve the exposure stability of the model in different application scenarios. Specifically, the SC module can be represented as:

$$G\;(l^{t}):\{\begin{array}{l} z^{t}=i\emptyset l^{t},\\ s^{t}=\kappa_{\vartheta }(z^{t}),\\ v^{t}=i+s^{t}, \end{array}$$ (3)

where *l*^*t*^ is the illumination at stage t; ∅ denotes division by elements; *κ*_*ϑ*_ is the parameter learnable operator; *s*^*t*^ is the self-calibrated mapping at moment *t*; and *v*^*t*^ is calibrated for the next stage of inputs. Fig 5 illustrates the calibration process in detail.

### 2.2 Loss function

The loss function of MTIE-Net consists of three parts [15, 17]: reconstruction loss $L_{r}$, denoising loss $L_{d}$, and illumination loss $L_{i}$, which can be expressed as follows:

$$L=L_{r}+L_{d}+L_{i}$$ (4)

In Retinex theory, the reflectance map is computed by a pixel-by-pixel division between the input image and its lighting map mapping, which serves as a constraint on the lighting map and the reflectance map [39], the reconstruction loss $L_{r}$ can be expressed as follows:

$$L_{r}={\left\|I-(R⊗L)\right\|}_{1}+{\left\|L-L0\right\|}_{1}+{\left\|R-I/L\right\|}_{1}$$ (5)

where *L*_*0*_ is the initial estimated R, G, and B channel intensity maximum of the lighting map.

The primary task of EDD-Net is to recover clean signals from reflectivity maps, thus the loss function should be able to quantify the difference between the network output and the true clean signal. In this paper, smoothing L1 loss is chosen as the loss function of EDD-Net. As a balanced form of L1 loss and L2 loss, it performs better for handling both noisy data and outliers. Smoothing L1 loss adds a smoothing parameter *ε*^*2*^ to the L2 loss, which is used to increase the stability of the values so that they do not suffer excessively as the L2 loss approaches zero. The denoising loss $L_{d}$ can be expressed as:

$$L_{d}=\frac{1}{N}\sum_{j=1}^{N}\sqrt{\left({\left(R_{nor}^{j}-{\hat{R}}_{low}^{j}\right)}^{2}+\varepsilon^{2}\right)}$$ (6)

Where *N* is the number of samples, the index *j* ∈ [1, *N*] represents the *j* th sample used to calculate the loss, which is consistent in the following equations. $R_{nor}^{j}$ is the reflectance map of the reference image, and ${\hat{R}}_{low}^{j}$ is the reflectance map of the low-light image after enhancement.

PCE-Net actually uses FFT and its inverse transform to realize the input image in the "spatial domain—frequency domain—spatial domain" between the conversion, and then achieve the purpose of illumination enhancement and detail reconstruction. Therefore, we use frequency loss [15] to enhance the performance of PCE-Net, and the frequency loss $L_{fre}$ can be expressed as:

$$L_{fre}=\frac{1}{N^{2}}\sum_{j=1}^{N}inf_{\gamma }∼\prod ({\hat{I}}_{low}^{j},I_{nor}^{j})E_{\left(x,y\right)}∼[\left\|{\hat{I}}_{low}^{j}‐I_{nor}^{j}\right\|]$$ (7)

Where in*f*_*γ*_~ denotes joint distribution. *E*_(*x*,*y*)_~ denotes using the Wasserstein distance to minimize the difference between the real part and imaginary part of enhanced result and the ground truth in frequency domain. ${\hat{I}}_{low}^{j}$ denotes the denoised low-light image, $I_{nor}^{j}$ denotes the denoised reference image.

In addition, we use the VGG loss to assist in the construction of the illumination loss, which is used to enhance the perceived quality of the enhanced image, and the VGG loss $L_{vgg}$ can be expressed as:

$$L_{vgg}=\frac{1}{N}\sum_{j=1}^{N}\left({\left\|\phi_{j}({\hat{R}}_{low}^{j})‐\phi_{j}(R_{nor}^{j})\right\|}^{2}\right)$$ (8)

Where *ϕ*_*j*_ denotes the loss network.

Then the illumination loss $L_{i}$ can be expressed as:

$$L_{i}=L_{fre}+L_{vgg}$$ (9)

**Fig 6:**
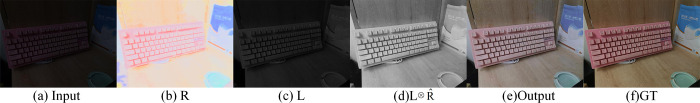
MTIE-Net processing and results.

As illustrated in Fig 6, combining $L_{r}$, $L_{d}$ and $L_{i}$, MTIE-Net can converge to decompose the input image *I* into a reflectance map *R* and lighting map *L*; Denoising the reflectance map and multiplying it element by element with the lighting map yields Fig 6(D); The final result is then enhanced to obtain Fig 6(E) and 6(F) shows the referenced normal light image.

## 3 Experiments

### 3.1 Implementation details

We use the publicly available dataset LSRW [17] as the training set. The performance of the model was validated using the test set of LSRW (containing 30 images), 100 randomly selected images from LOL [15], and 15 randomly selected images from the MIT-5k dataset as three independent test sets, respectively. The experimental environment configuration is shown in Table 1. For model training, the epoch is set to 40; the batch size is 4; and the patch size is 96; using the Adam [40] optimizer, the initial learning rate is set to 10^−3^, and after 20 rounds of training the learning rate is reduced to 10^−4^ according to the learning rate decay strategy.

**Table 1:** Experimental setting.

Software/Hardware	Models
CPU	AMD EPYC 7302
GPU	Nvidia Ampere A100
Deep learning framework	Pytorch
Python version	3.8

### 3.2 Evaluation metrics

In this paper, image quality assessment metrics such as peak signal-to-noise ratio (PSNR) [41], structural similarity (SSIM) [42], learned perceptual image patch similarity (LPIPS) [43], natural image quality evaluator (NIQE) [44], average gradient (AG) and spatial frequency (SF) are used to evaluate the performance of MTIE-Net. In which, PSNR, SSIM, LPIPS are full reference metrics and NIQE, AG, SF are no reference metrics. PSNR evaluates the image quality by calculating the pixel error in the global range, and the larger its value, the lower the distortion and the higher the quality of the enhanced image. SSIM combines the brightness, contrast and structural information similarity between two images to assess the quality of the processed image relative to the GT image, the size of the value between 0 and 1, the closer the value of 1 indicates that the enhanced image is more similar to the original image, the better the enhancement effect. LPIPS calculates the perceptual difference between the enhanced image and the GT image by establishing an inverse mapping relationship between the two images, with lower values indicating that the two images are more similar. NIQE predicts the perceived quality of an image by analyzing the statistical properties of the natural scene of the image, with lower values indicating that the enhanced image is of higher quality and closer to the natural image. AG is used as a measure of image sharpness, the larger the AG, the sharper the enhanced image. SF reflects the rate of change of the gray scale of the image, the larger value indicates that the image is clearer, the better the quality of the enhanced image.

### 3.3 Ablation study

#### 3.3.1 PMA structural ablation study

Firstly, we conduct an ablation study on the LOL dataset to validate the effectiveness of PMA and the optimal way of integrating the three attentional mechanisms of CAM, SAM, and PAM, using PSNR and SSIM as objective evaluation metrics.

**Fig 7:**
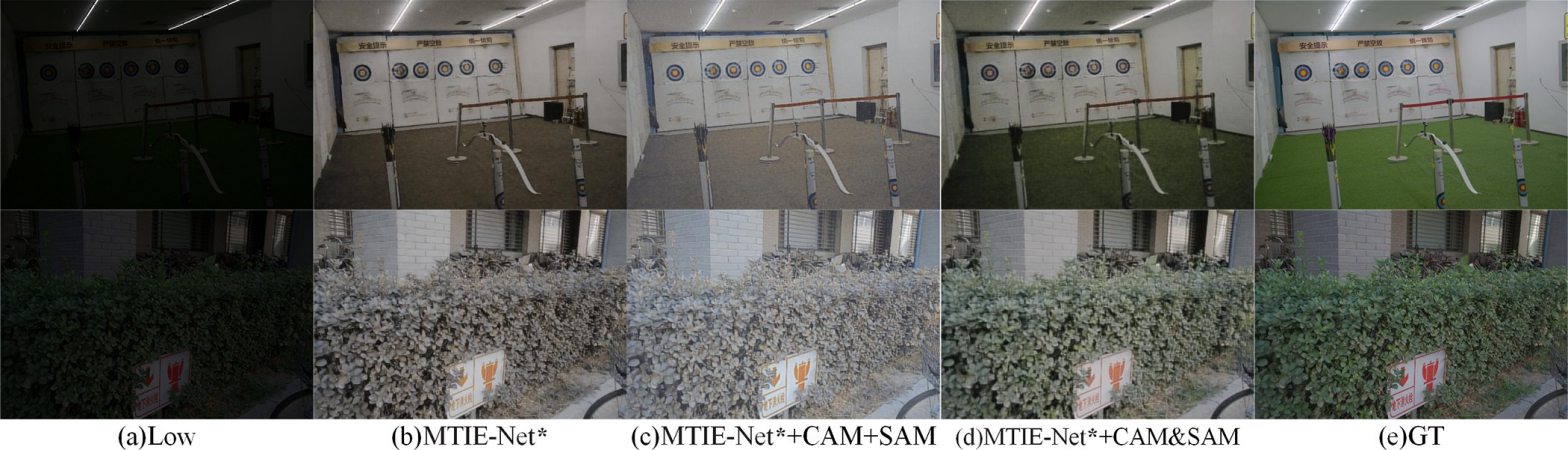
Comparison of subjective enhancement effect of different combined structures of CAM and SAM.

As shown in Table 2: connecting the PAM serially to the parallel structure after the CAM and the SAM, the network obtains the highest PSNR and SSIM values, achieving the best enhancement results in comparison (MTIE-Net * denotes that the network has not added any of the modules of RDB, CTrans, PMA and SC; "+" denotes the serial structure and "&" denotes the parallel structure). Additionally, we randomly select two images from the LOL dataset for the enhancement test to further verify the superiority of the parallel structure of CAM and SAM. The comparison of subjective effects is illustrated in Fig 7.

**Table 2:** The ablation experiment of PMA structure.

Models	PSRN	SSIM
MTIE-Net*	17.887	0.741
MTIE-Net* + CAM + SAM	20.001	0.758
MTIE-Net* + CAM & SAM	20.033	0.765
MTIE-Net* + PAM + CAM + SAM	19.979	0.762
MTIE-Net* + CAM + SAM + PAM	19.934	0.755
MTIE-Net* + PAM + CAM & SAM	20.082	0.768
MTIE-Net* + CAM & SAM + PAM	**20.250**	**0.771**

#### 3.3.2 MTIE-Net compositional ablation study

**Fig 8:**
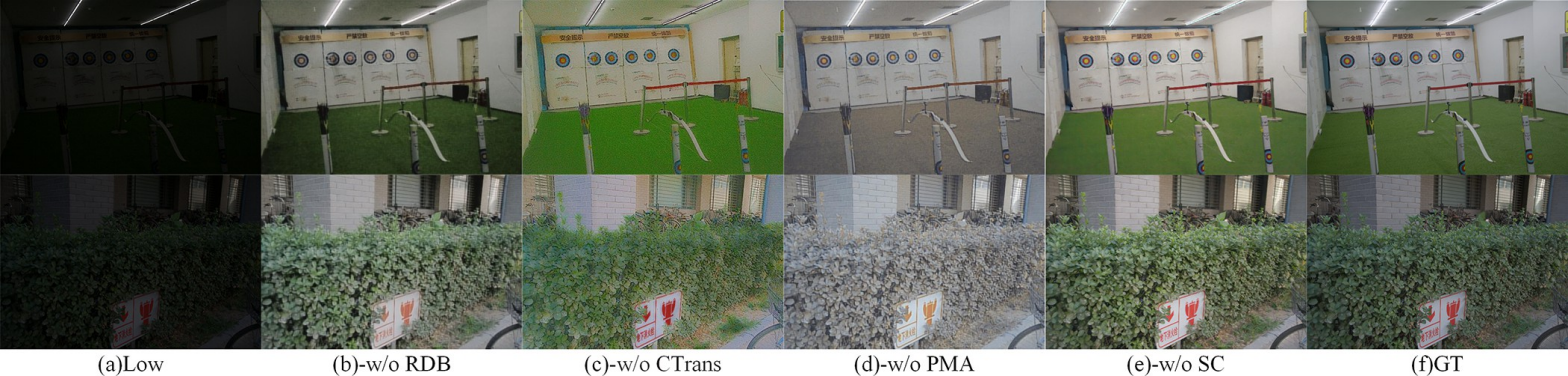
Qualitative results of ablation experiments.

Further, to comprehensively demonstrate the positive impact of each core module in MTIE-Net, we performed ablation studies in the LOL dataset. Specifically, 1) MTIE-Net without residual dense block (-w/o RDB), 2) MTIE-Net without channel transformer (-w/o CTrans), 3) MTIE-Net without Pixel attention module (-w/o PMA), 4) MTIE-Net without self-calibrated (-w/o SC). Fig 8 can observe the visual results of the ablation experiments: (b) -w/o RDB does not reduce noise; (c) -w/o CTrans does not enhance the texture structure of images, (d) -w/o PMA does not remove color distortion, (e) -w/o SC does not improve contrast. The average scores of PSNR and SSIM for the ablation model are given in Table 3, which shows that our full model obtained the highest score. Meanwhile, it is further proved that each core module positively affects MTIE-Net.

**Table 3:** Quantitative results of ablation experiments.

Model	PSRN	SSIM
MTIE-Net*	17.887	0.741
-w/o RDB	20.271	0.772
-w/o CTrans	20.109	0.769
-w/o PMA	19.874	0.766
-w/o SC	20.283	0.775
MTIE-Net (full model)	**20.409**	**0.783**

#### 3.3.3 Calculation cost analysis

Finally, we validate the computational efficiency of our MTIE-Net as a way to demonstrate the positive impact of the self-calibrated training strategy in reducing the computational effort and speeding up the convergence of the network. Table 4 compares the FLOPs and running time (GPU-seconds for inference) of MTIE-Net with some state-of-the-art models. To ensure the rigor of the experiments, we randomly selected 100 test images of size 600*400 from the LOL dataset and calculated their FLOPs and running time in the Pytorch platform (GPU). As shown in Table 4, the FLOPs and runtime of MTIE-Net are significantly reduced after the introduction of the self-calibrated training strategy. While the computational cost of MTIE-Net is higher than SCI, notably, it is a complete network that allows for simultaneous denoising and enhancement. In conclusion, Table 4 can fully verify the efficiency and rapidity of MTIE-Net. The best result is in red whereas the second best one is in blue.

**Table 4:** Comparison of calculation costs.

Method	FLOPs	running time
RetinexNet	136.0155	0.1198
RUAS	0.2871	0.0063
SCI	**0.0724**	**0.0021**
ZERO-DCE	5.2132	0.0051
MTIE-Net (-w/o SC)	8.2738	0.0618
MTIE-Net (full model)	*2*.*8102*	*0*.*0045*

### 3.4 Comparison experiment

We compare MTIE-Net with RetinexNet, R2R, RUAS [45], SNR, ZERO-DCE, and SCI on three publicly available datasets.

As shown in Table 5: MTIE-Net outperforms the comparison methods in SSIM, LPIPS, NIQE, AG and SF on the LOL dataset. The average SSIM value is 0.783, the average LPIPS value is 0.343, the average NIQE value is 3.905, the average AG value is 10.321 and the average SF value is 11.788; the average PSNR value is 20.409 dB, which is only 0.067 dB lower than the SNR with the highest average value. On the LSRW dataset, the PSNR, SSIM, LPIPS, AG and SF of MTIE-Net outperform the comparison methods. The average PSNR value was 21.575, the average SSIM value was 0.801, the average LPIPS value was 0.336, the average AG value was 4.675 and the average SF value was 7.692; the average NIQE value was 3.921, which was only 0.101 higher than the ZERO-DCE, with the lowest average value. On the MIT-5k dataset, MTIE-Net achieved the lowest NIQE values; the average AG value is 3.247, which is only 0.052 lower than the ZERO-DCE with the highest average value; the average SF value is 5.810, which is only 0.122 lower than the ZERO-DCE with the highest average value. In summary, it is shown that MTIE-Net achieves the best enhancement performance on both LOL and LSRW datasets. Comprehensive performance is also at the top of the MIT-5k dataset.

**Table 5:** Comparison of objective evaluation metrics.

Datasets	Metrics	Method						
		RetinexNet	R2R	RUAS	SNR	ZERO-DCE	SCI	Ours
LOL	PSNR	15.720	17.892	14.846	**20.476**	18.059	17.253	*20*.*409*
	SSIM	0.468	0.752	0.490	*0*.*774*	0.580	0.551	**0.783**
	LPIPS	0.589	0.395	0.434	*0*.*351*	0.361	0.355	**0.343**
	NIQE	4.712	*4*.*240*	7.695	4.733	7.513	7.745	**3.905**
	AG	3.959	4.119	*6*.*615*	4.322	6.258	6.323	**10.321**
	SF	6.175	6.852	10.008	6.649	*10*.*424*	10.075	**11.788**
LSRW	PSNR	15.090	17.267	15.687	*17*.*614*	16.396	15.090	**21.575**
	SSIM	0.415	0.540	0.481	*0*.*578*	0.465	0.415	**0.801**
	LPIPS	*0*.*445*	0.476	0.552	0.502	0.451	*0*.*445*	**0.336**
	NIQE	4.035	4.408	5.800	7.546	**3.820**	4.035	*3*.*921*
	AG	2.517	2.664	2.504	3.933	*3*.*843*	3.826	**4.675**
	SF	4.865	5.055	4.057	6.498	*6*.*978*	6.826	**7.692**
MIT-5k	PSNR	14.198	17.591	18.714	17.560	16.664	**19.907**	*18*.*944*
	SSIM	0.727	0.774	0.758	0.652	*0*.*780*	**0.834**	0.761
	LPIPS	0.516	*0*.*407*	0.453	0.574	0.443	**0.327**	0.416
	NIQE	4.529	4.261	4.587	5.133	4.101	*3*.*963*	**3.904**
	AG	1.673	2.981	3.598	1.117	**3.299**	3.184	*3*.*247*
	SF	3.657	4.824	5.297	2.649	**5.932**	5.289	*5*.*810*

Additionally, in order to further verify the superiority of MTIE-Net enhancement performance, we randomly selected two low-light images from each of the LOL and LSRW datasets to test the enhancement effect of the above comparison algorithms.

**Fig 9:**
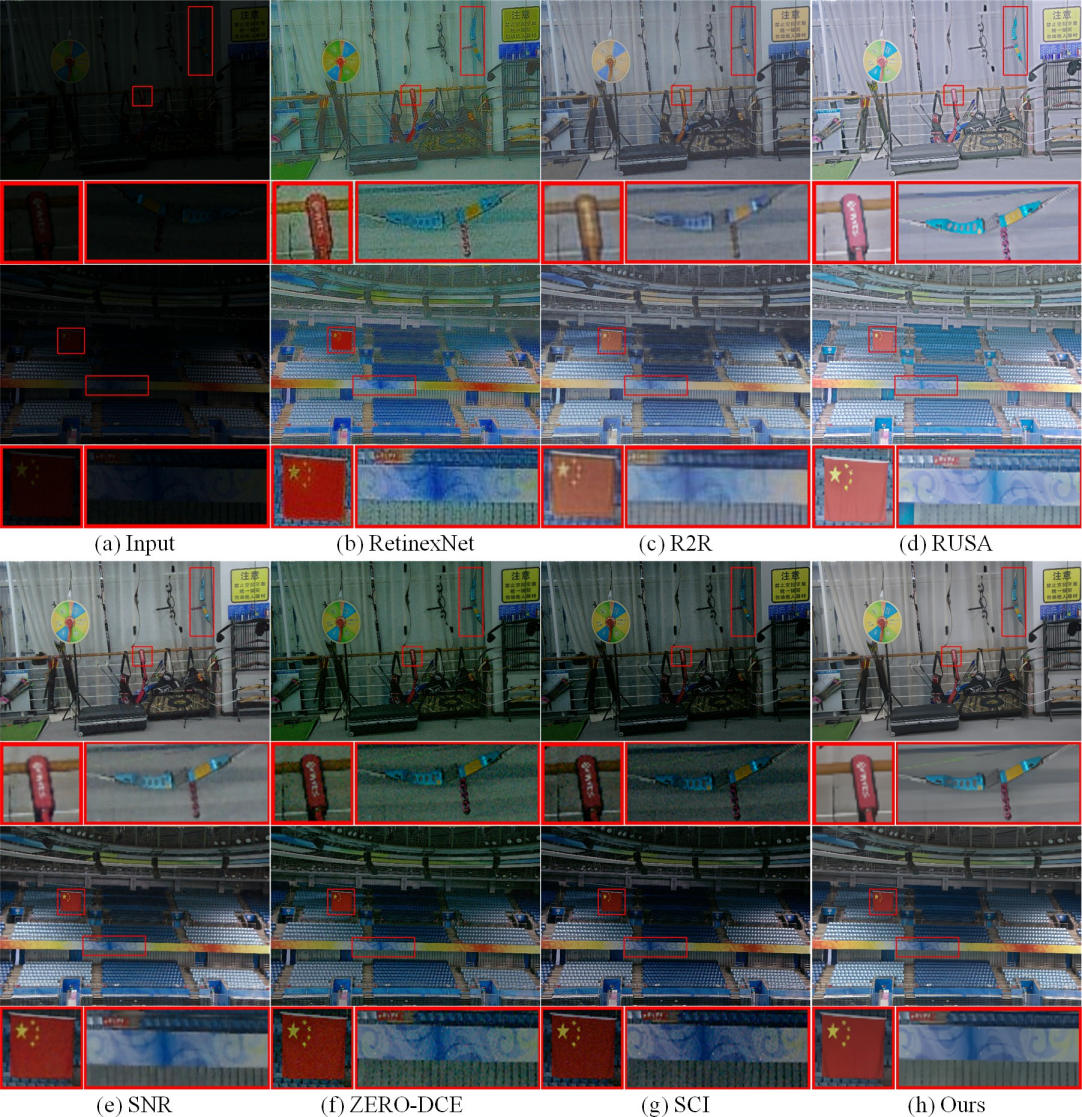
Comparison of enhancement effects on the LOL dataset.

**Fig 10:**
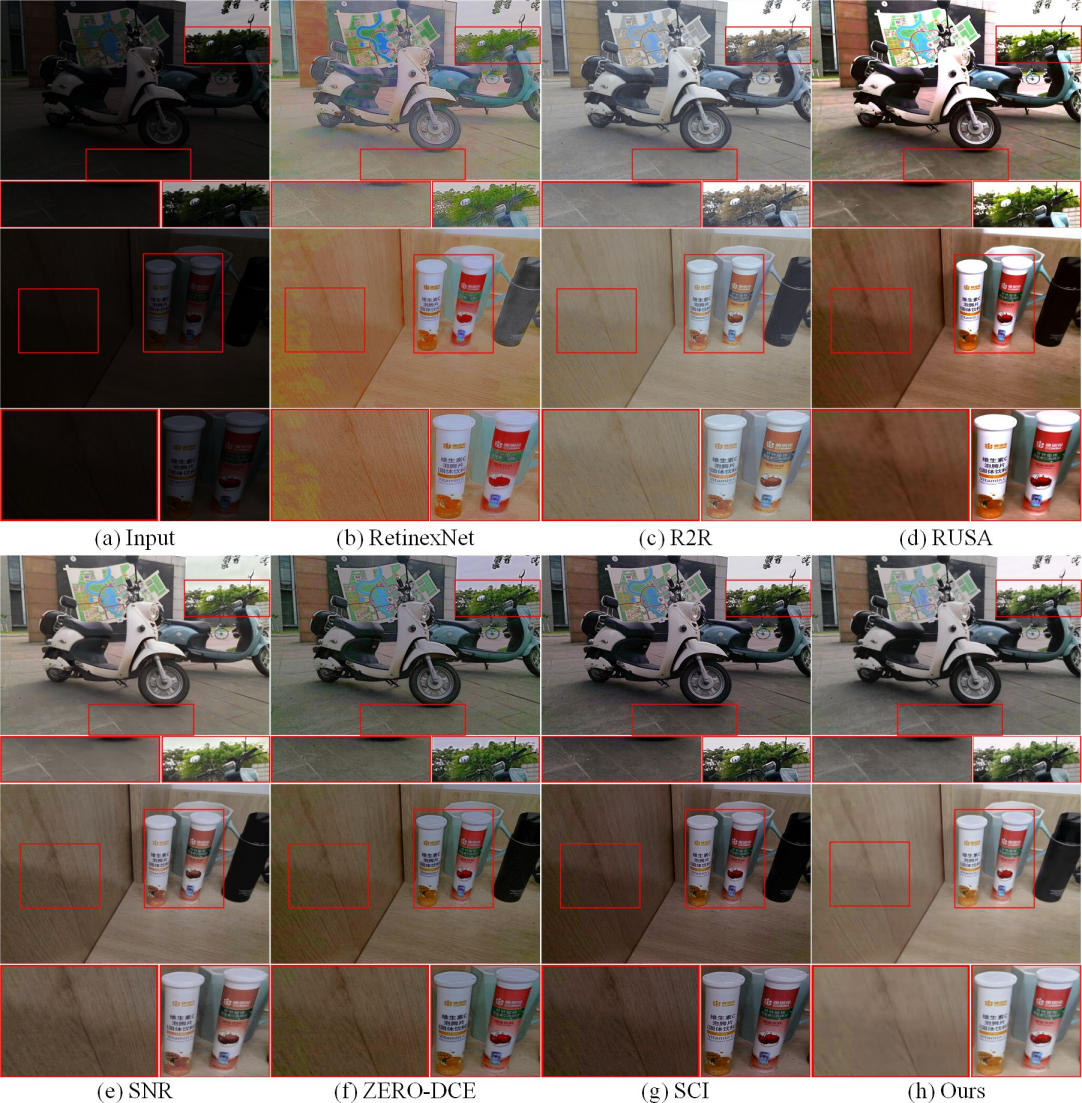
Comparison of enhancement effects on the LSRW dataset.

As demonstrated in Figs 9 and 10, RetinexNet exhibits detail blurring and noise amplification on both datasets; R2R showed significant color distortion problems on both datasets; RUAS showed varying degrees of overexposure on both datasets, while ZERO-DCE and SCI showed significant underexposure, especially on the LOL dataset; Objects in SNR enhanced images have distinct colors, but the network is inferior to MTIE-Net in image detail and texture restoration. Detailed comparisons of the enhancement effects of each algorithm have been labeled with red wireframes in Figs 9 and 10. In summary, MTIE-Net shows significant enhancement performance across various low-light datasets. Compared to existing single enhancement techniques, MTIE-Net is able to enhance the luminance and preserve details of low-light images more effectively, while suppressing noise. A large number of comparison experiments in Table 4 also fully prove the effectiveness and superiority of MTIE-Net.

### 3.5 Pre-processing for face detection in low-light environments

**Fig 11:**
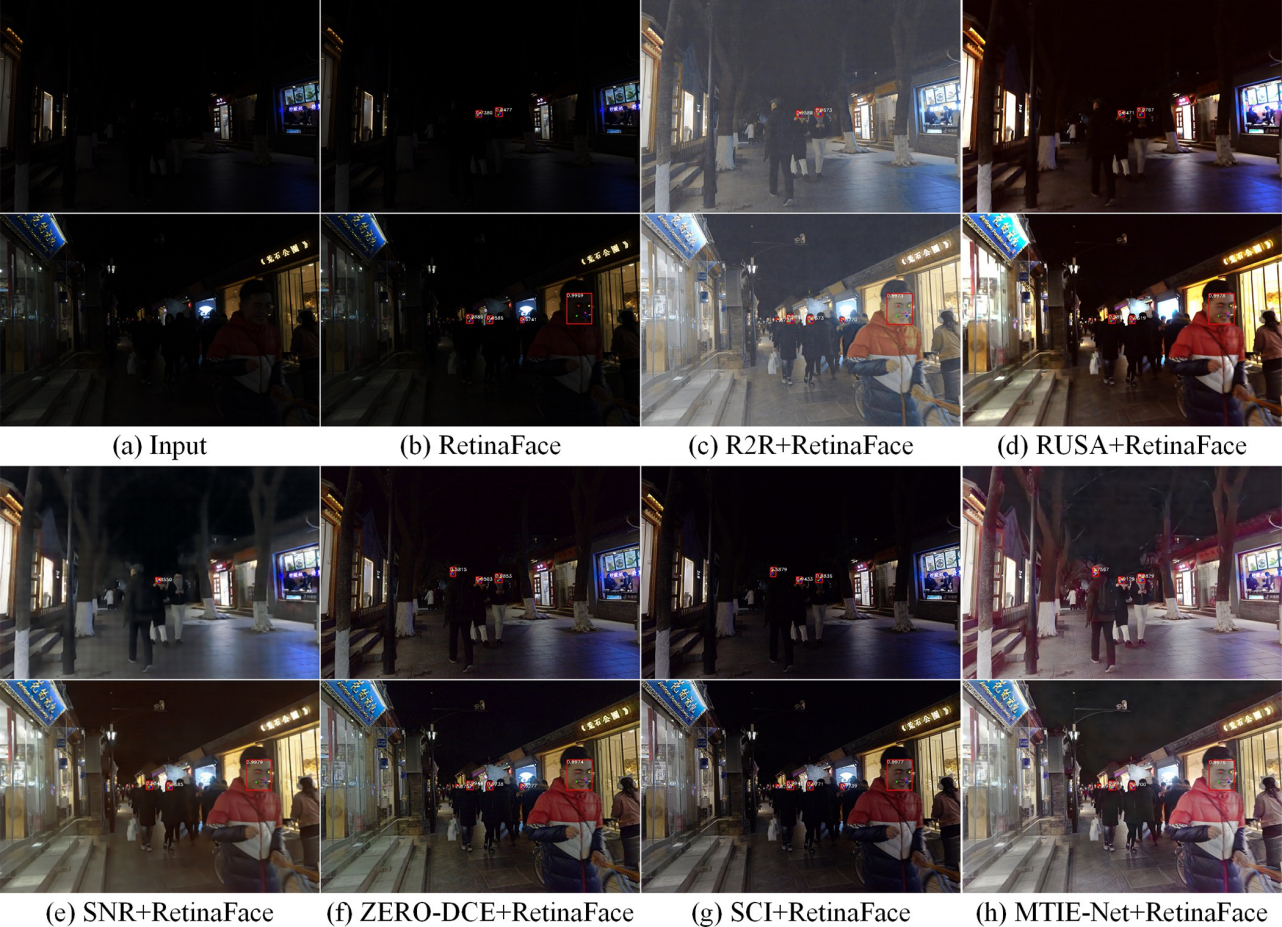
Comparison of nighttime face detection effect.

In this section, we compare MTIE-Net as a pre-processing tool for nighttime face detection with the algorithm described in Section 3.4, in order to verify its impact on advanced visual tasks. We use the current state-of-the-art face detection algorithm RetinaFace [46] in the DARK FACE [47] dataset for comparison experiments. As illustrated in Fig 11: MTIE-Net improves RetinaFace detection performance most significantly in low-light environments. The experimental results further validate the generalizability of MTIE-Net and also show its potential for application in advanced vision tasks.

## 4 Conclusion

In this paper, we propose a Multi-technology fusion of low-light image enhancement network (MTIE-Net). The network modularizes the low-light enhancement task into three parts: decomposition, denoising and enhancement based on Retinex theory. Our experiments on several publicly available datasets demonstrate MTIE-Net’s ability to effectively enhance visual quality and suppress noise in images. In addition, the nighttime face detection experiments demonstrate the promising application of MTIE-Net as a pre-processing means to enhance the performance of advanced visual tasks. Overall, this study presents a comprehensive and effective solution to low-light image enhancement, offering significant theoretical and practical value.

## Supporting information

S1 File Supporting information contains the study’s minimal underlying data set. (ZIP)
