# Supplementary figures and images for: MTIE-Net: Multi-technology fusion of low-light image enhancement network
Source: PLoS One. 2024 Feb 2;19(2):e0297984. doi: 10.1371/journal.pone.0297984 (PMC10836710; doi:10.1371/journal.pone.0297984)

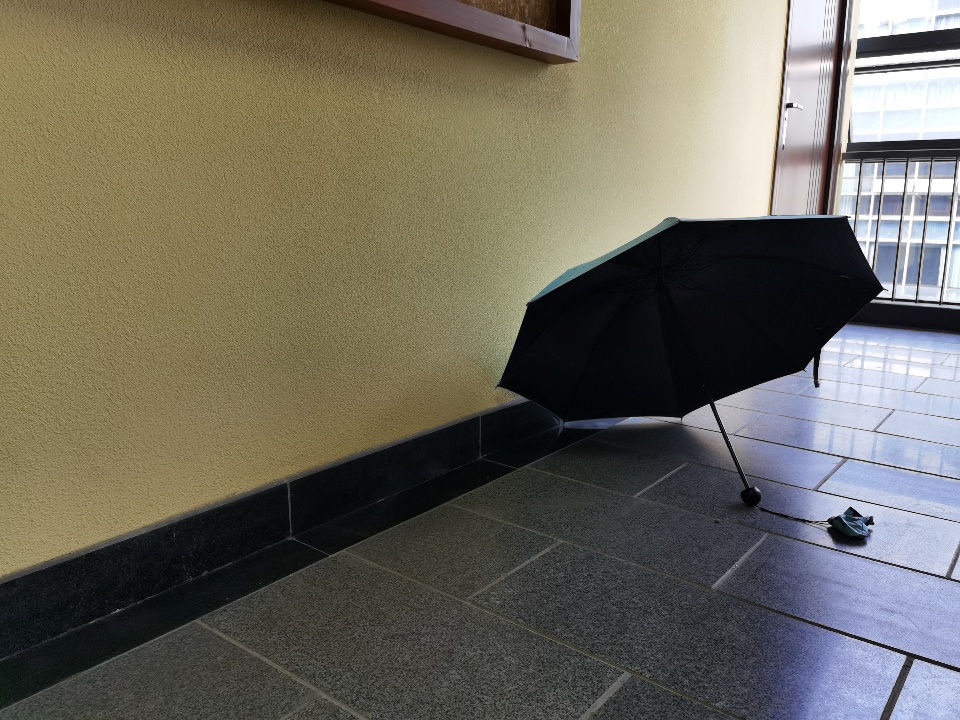

Supplement: S1 File — (ZIP) [file pone.0297984.s001.zip › EVAL/high/2037.jpg]

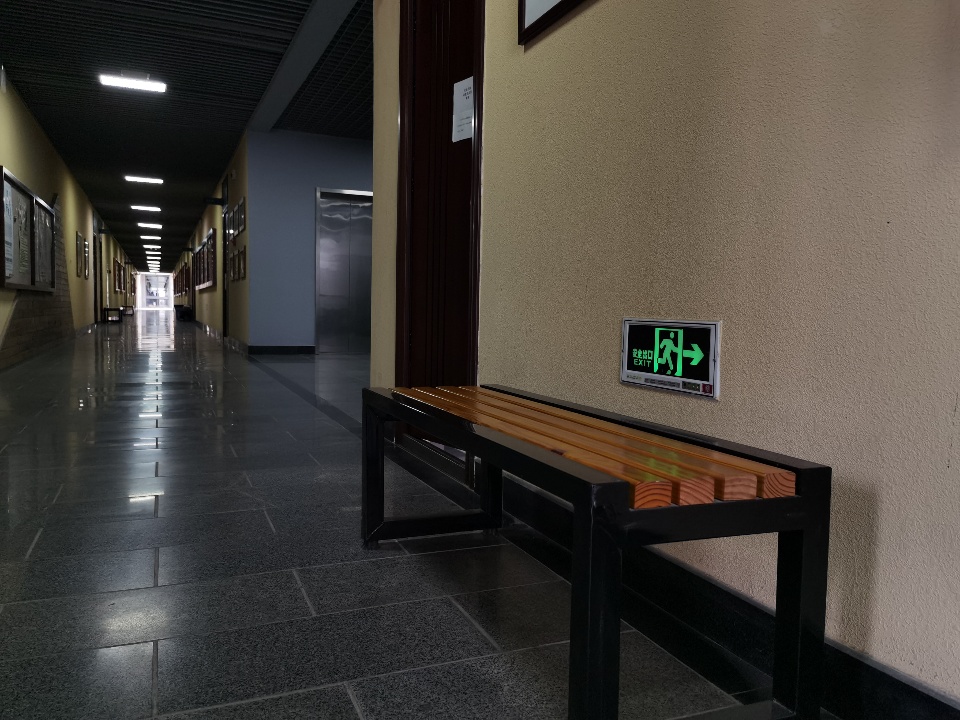

Supplement: S1 File — (ZIP) [file pone.0297984.s001.zip › EVAL/high/2038.jpg]

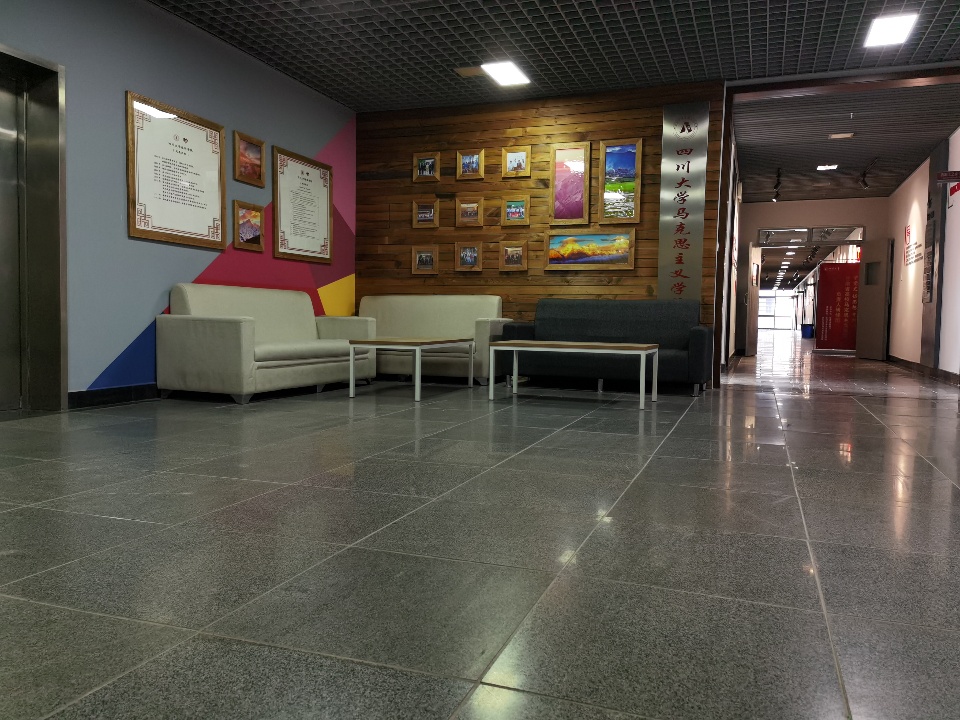

Supplement: S1 File — (ZIP) [file pone.0297984.s001.zip › EVAL/high/2039.jpg]

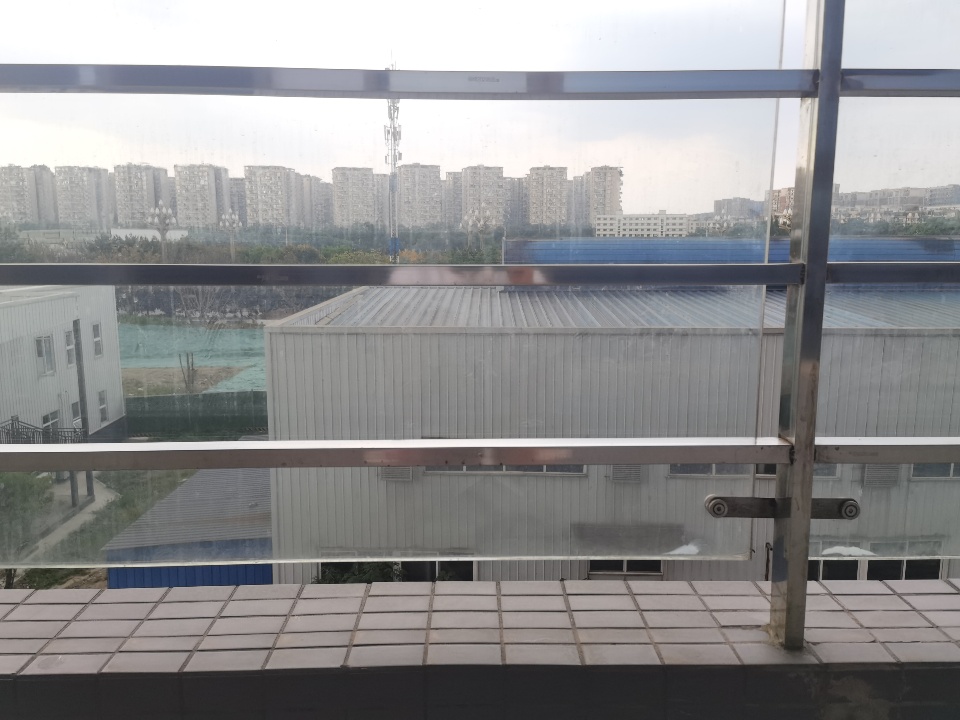

Supplement: S1 File — (ZIP) [file pone.0297984.s001.zip › EVAL/high/2040.jpg]

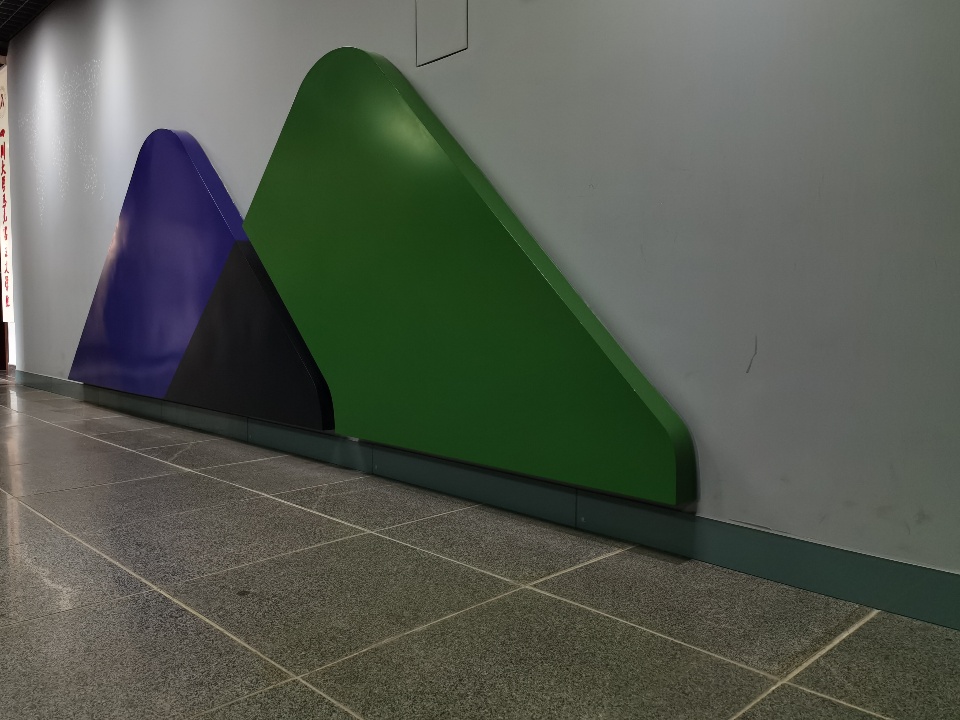

Supplement: S1 File — (ZIP) [file pone.0297984.s001.zip › EVAL/high/2041.jpg]

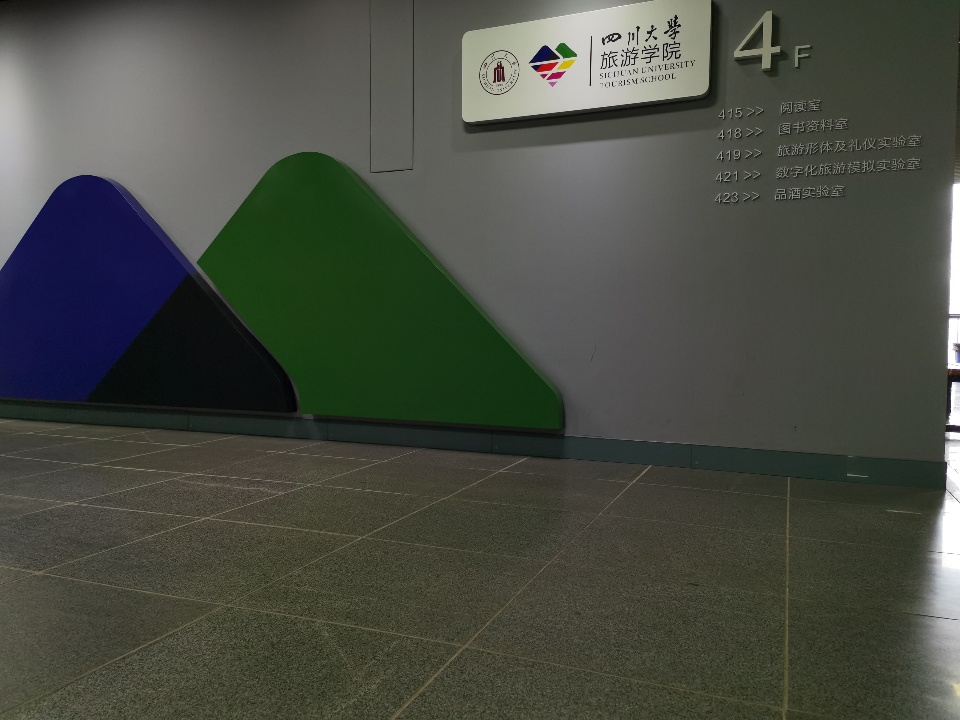

Supplement: S1 File — (ZIP) [file pone.0297984.s001.zip › EVAL/high/2042.jpg]

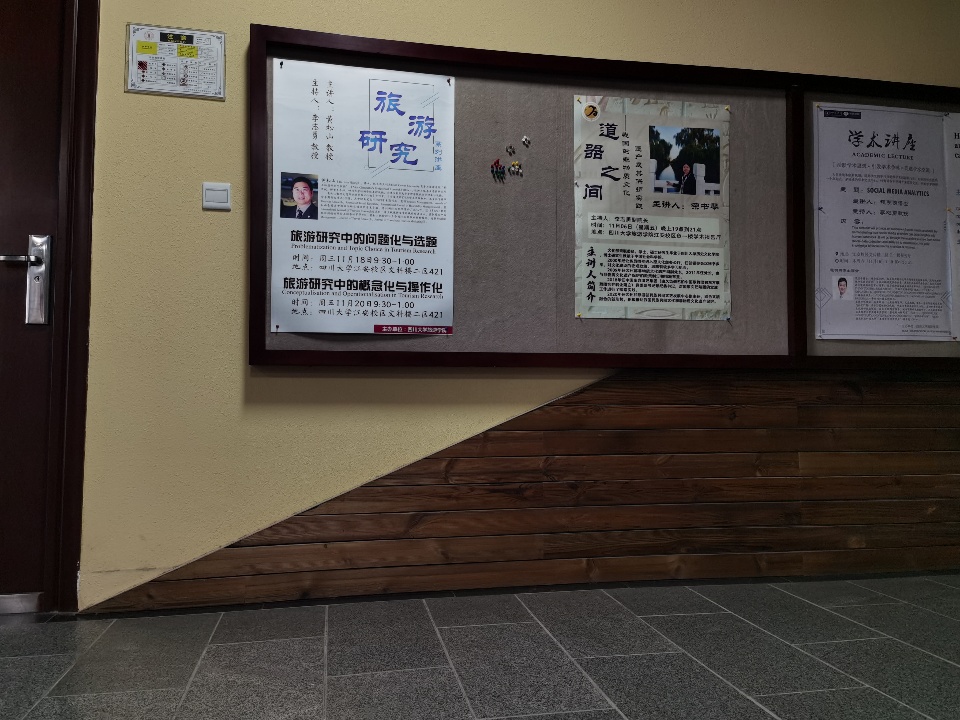

Supplement: S1 File — (ZIP) [file pone.0297984.s001.zip › EVAL/high/2043.jpg]

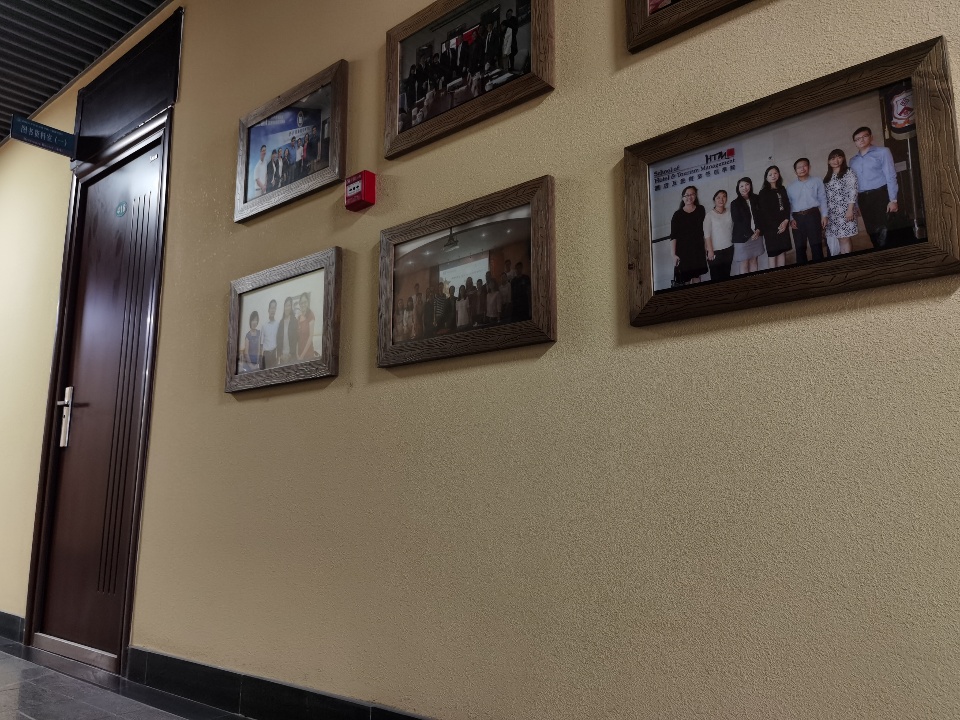

Supplement: S1 File — (ZIP) [file pone.0297984.s001.zip › EVAL/high/2044.jpg]

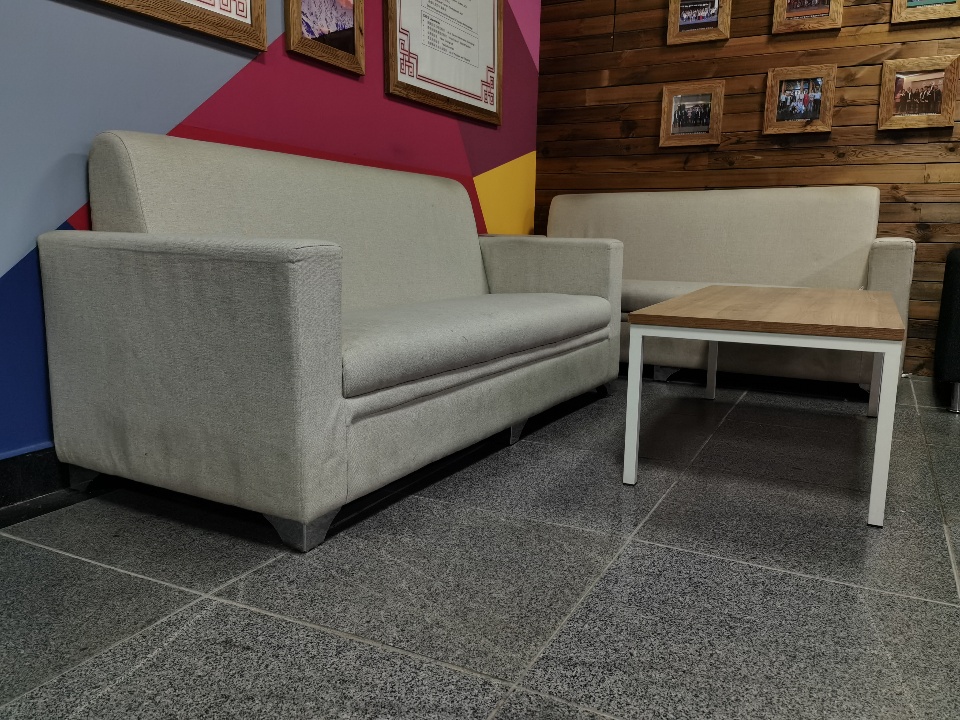

Supplement: S1 File — (ZIP) [file pone.0297984.s001.zip › EVAL/high/2045.jpg]

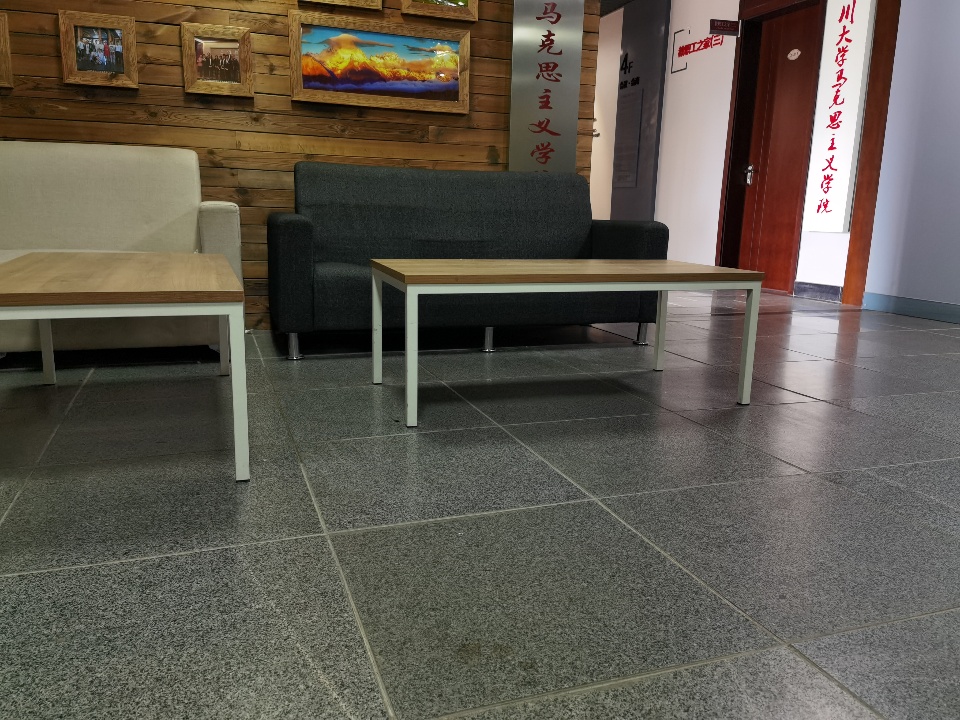

Supplement: S1 File — (ZIP) [file pone.0297984.s001.zip › EVAL/high/2046.jpg]

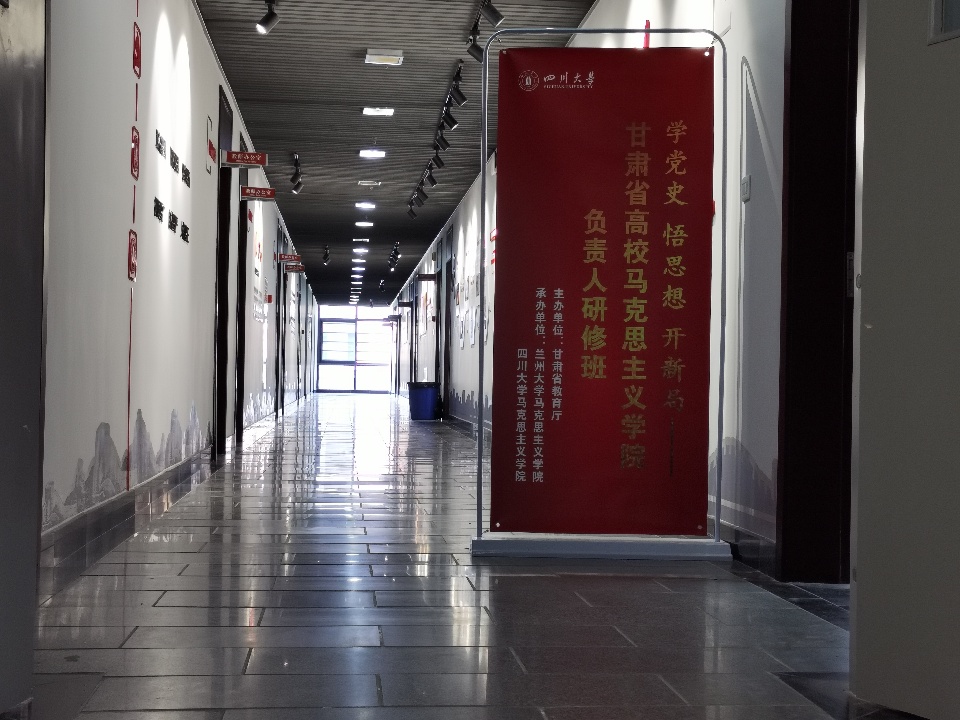

Supplement: S1 File — (ZIP) [file pone.0297984.s001.zip › EVAL/high/2047.jpg]

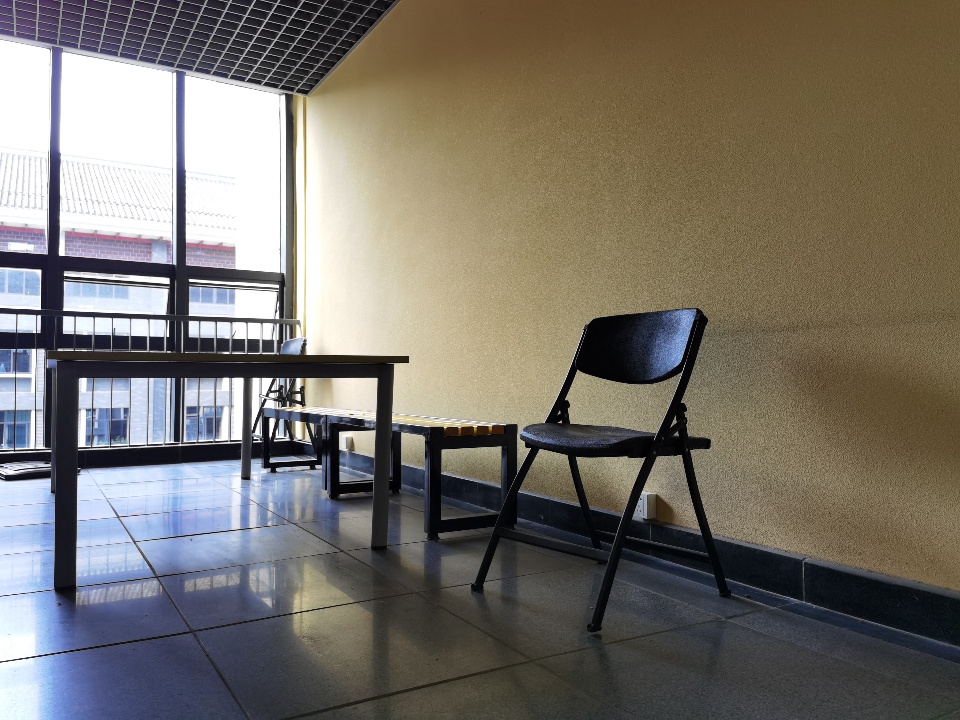

Supplement: S1 File — (ZIP) [file pone.0297984.s001.zip › EVAL/high/2048.jpg]

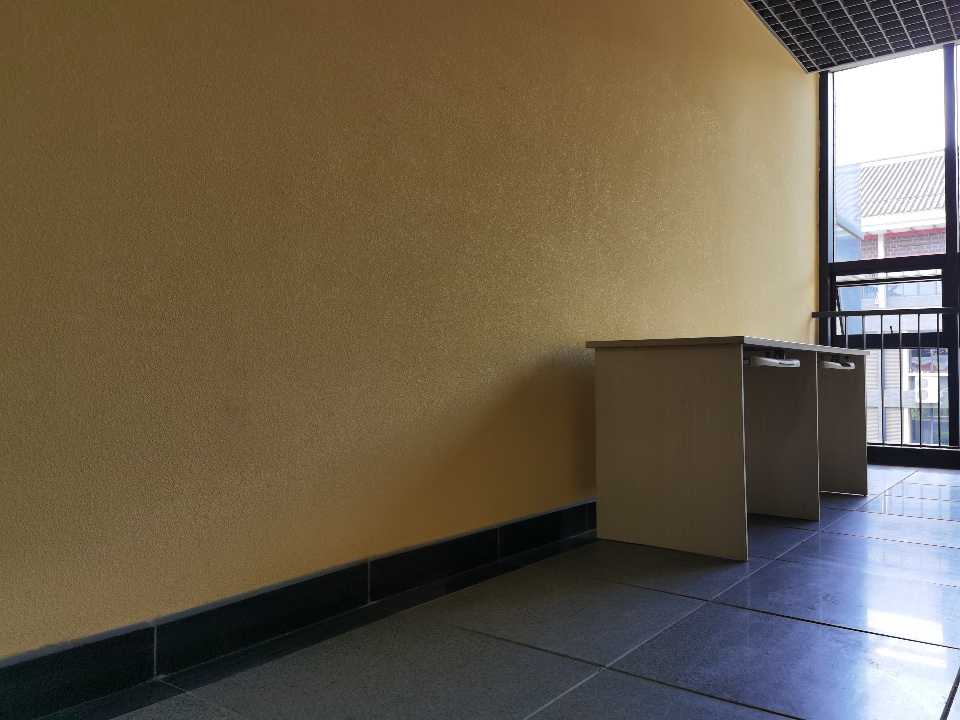

Supplement: S1 File — (ZIP) [file pone.0297984.s001.zip › EVAL/high/2049.jpg]

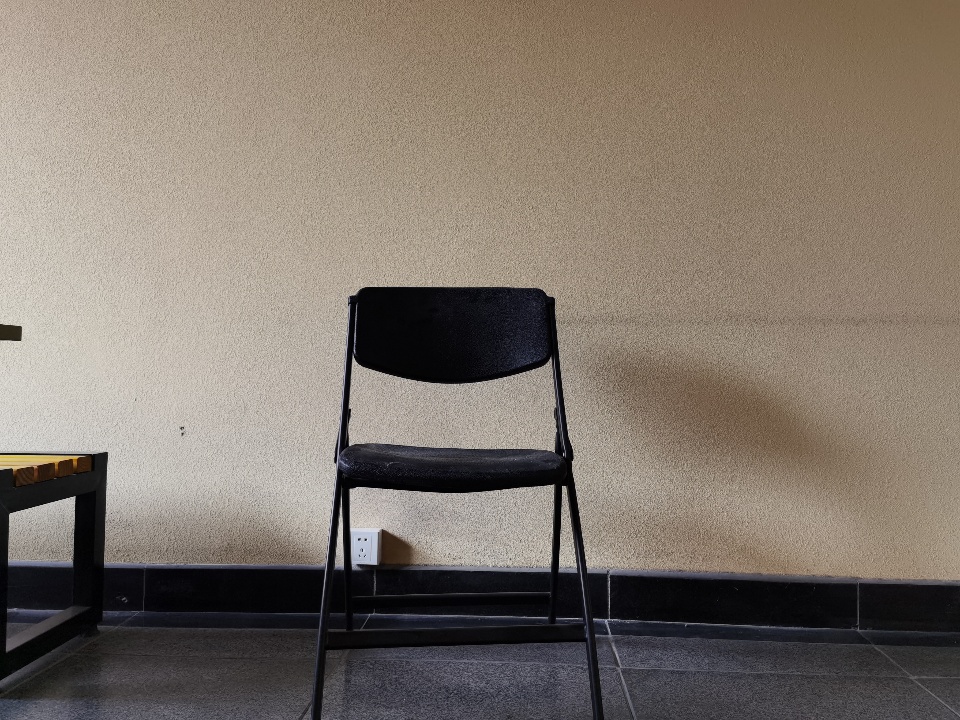

Supplement: S1 File — (ZIP) [file pone.0297984.s001.zip › EVAL/high/2050.jpg]

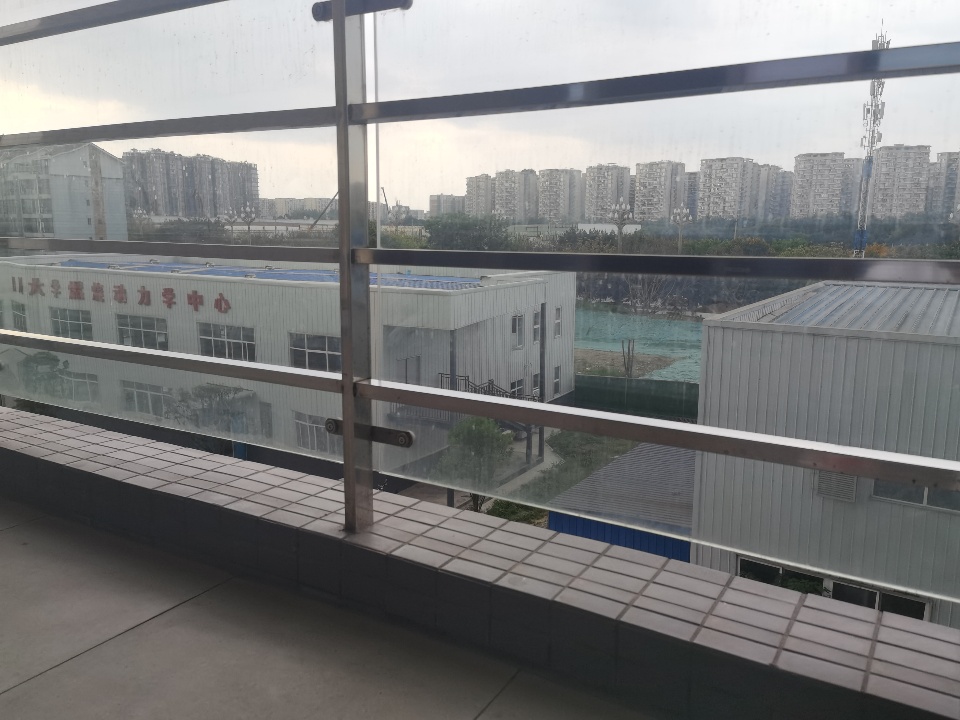

Supplement: S1 File — (ZIP) [file pone.0297984.s001.zip › EVAL/high/2051.jpg]

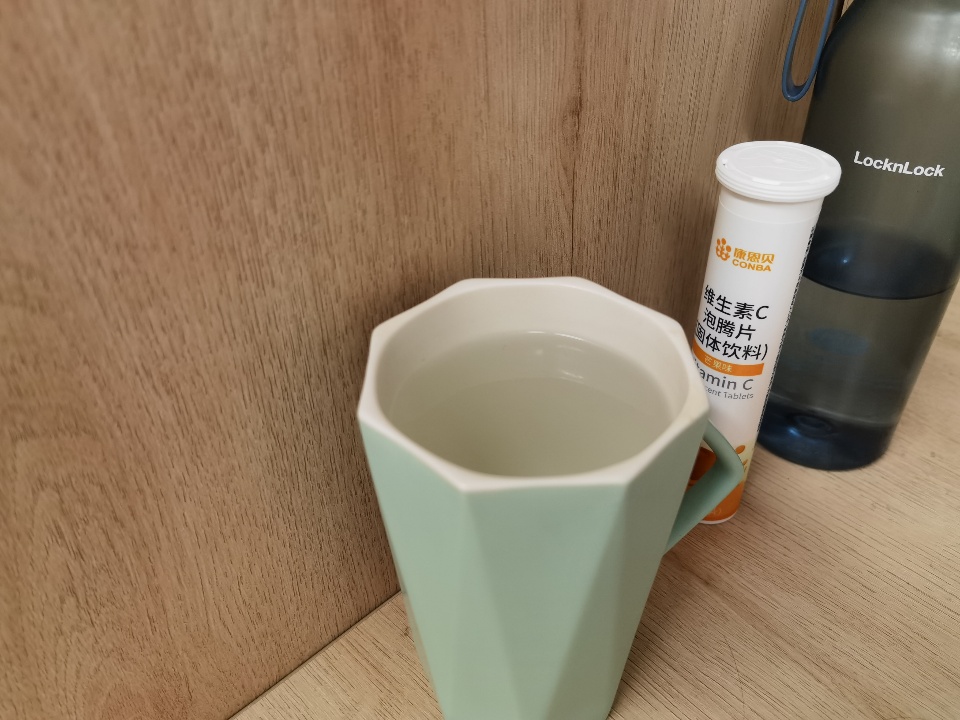

Supplement: S1 File — (ZIP) [file pone.0297984.s001.zip › EVAL/high/2052.jpg]

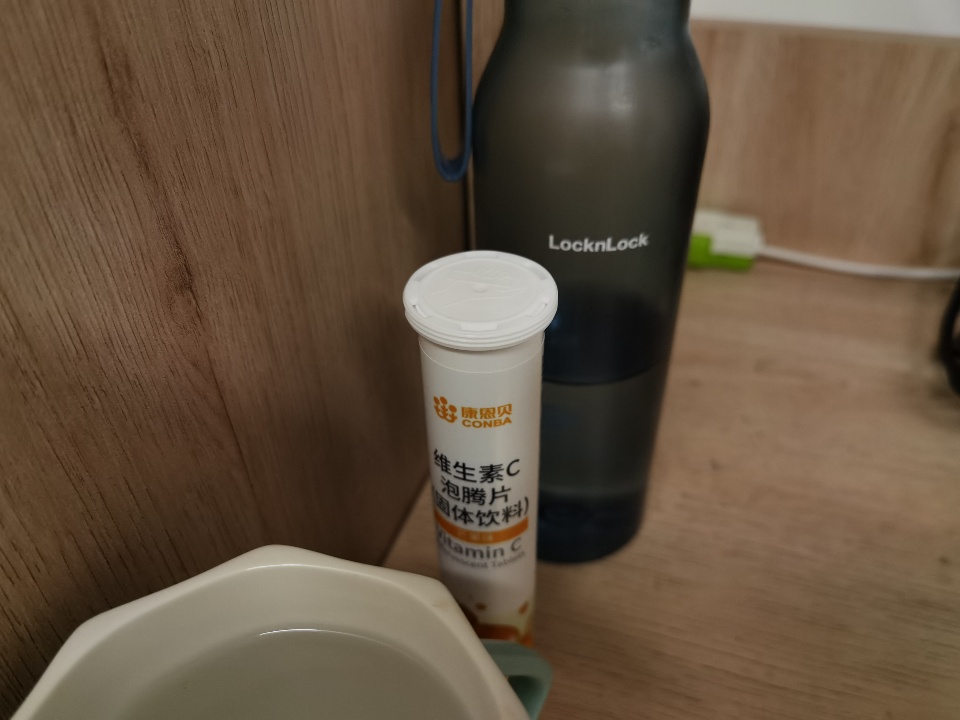

Supplement: S1 File — (ZIP) [file pone.0297984.s001.zip › EVAL/high/2053.jpg]

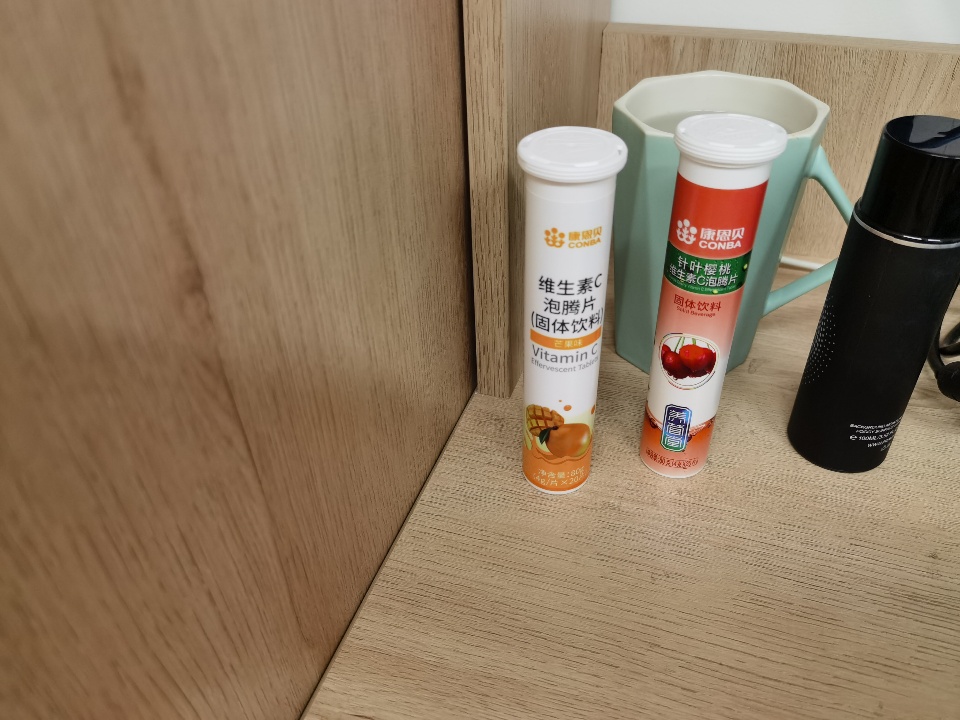

Supplement: S1 File — (ZIP) [file pone.0297984.s001.zip › EVAL/high/2054.jpg]

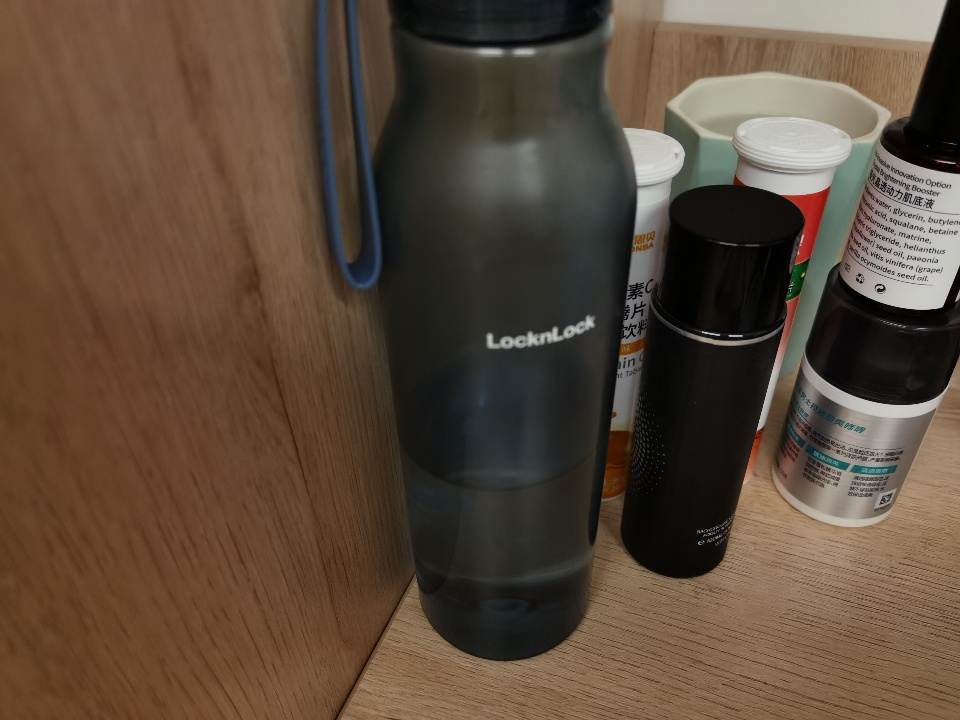

Supplement: S1 File — (ZIP) [file pone.0297984.s001.zip › EVAL/high/2055.jpg]

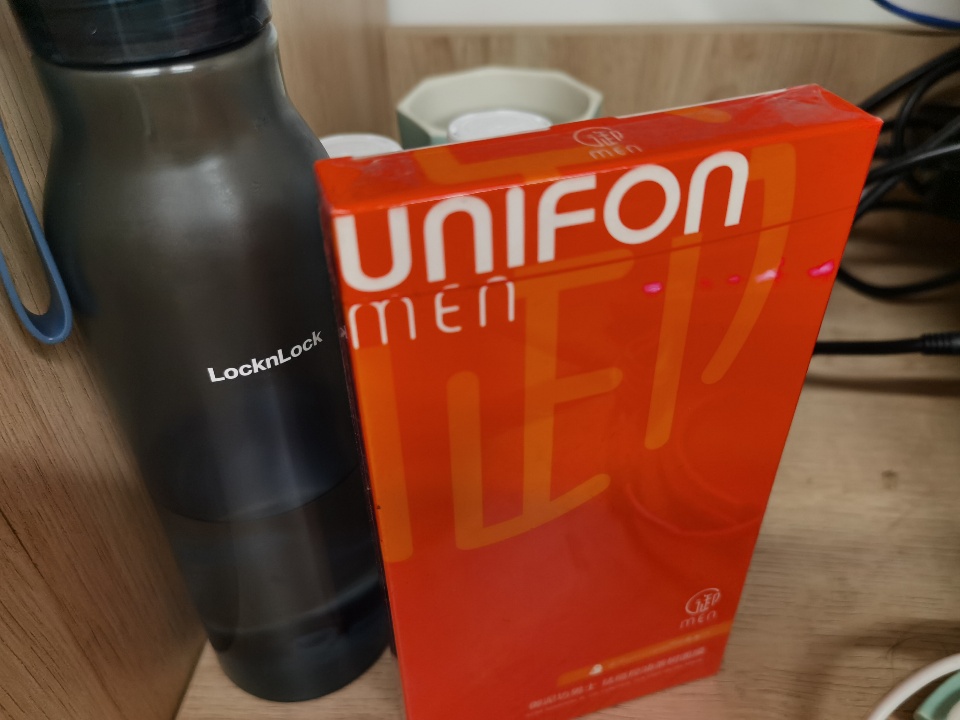

Supplement: S1 File — (ZIP) [file pone.0297984.s001.zip › EVAL/high/2056.jpg]

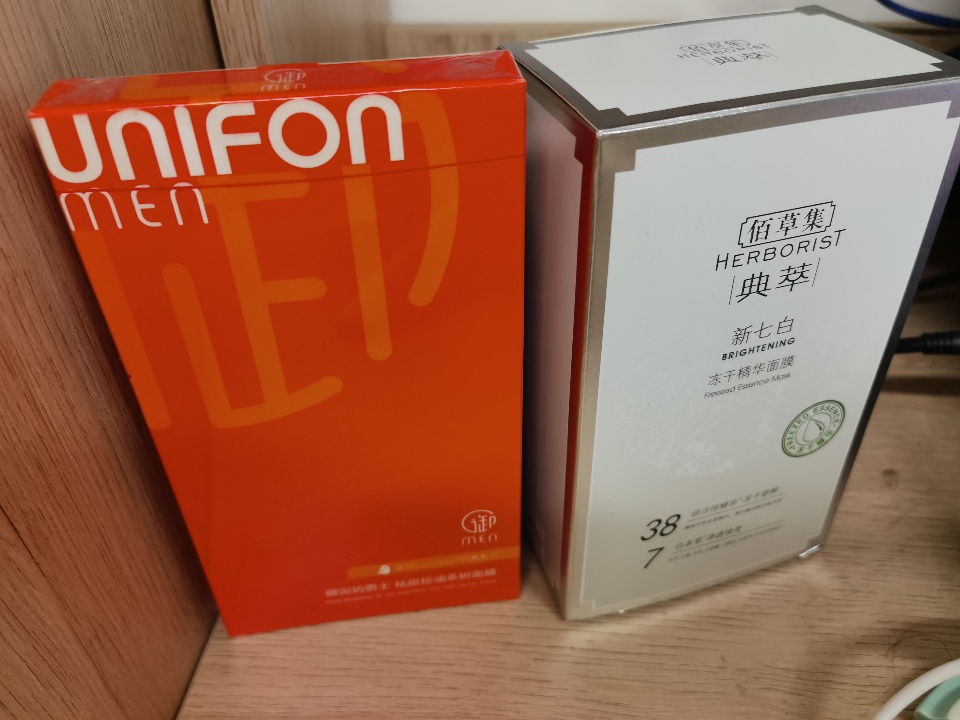

Supplement: S1 File — (ZIP) [file pone.0297984.s001.zip › EVAL/high/2057.jpg]

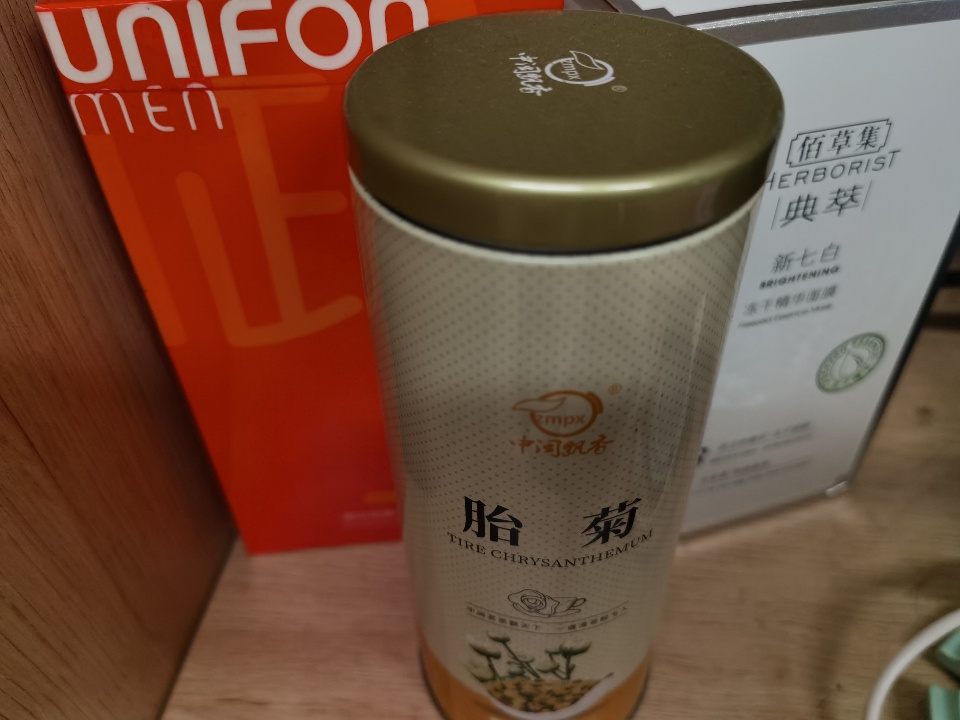

Supplement: S1 File — (ZIP) [file pone.0297984.s001.zip › EVAL/high/2058.jpg]

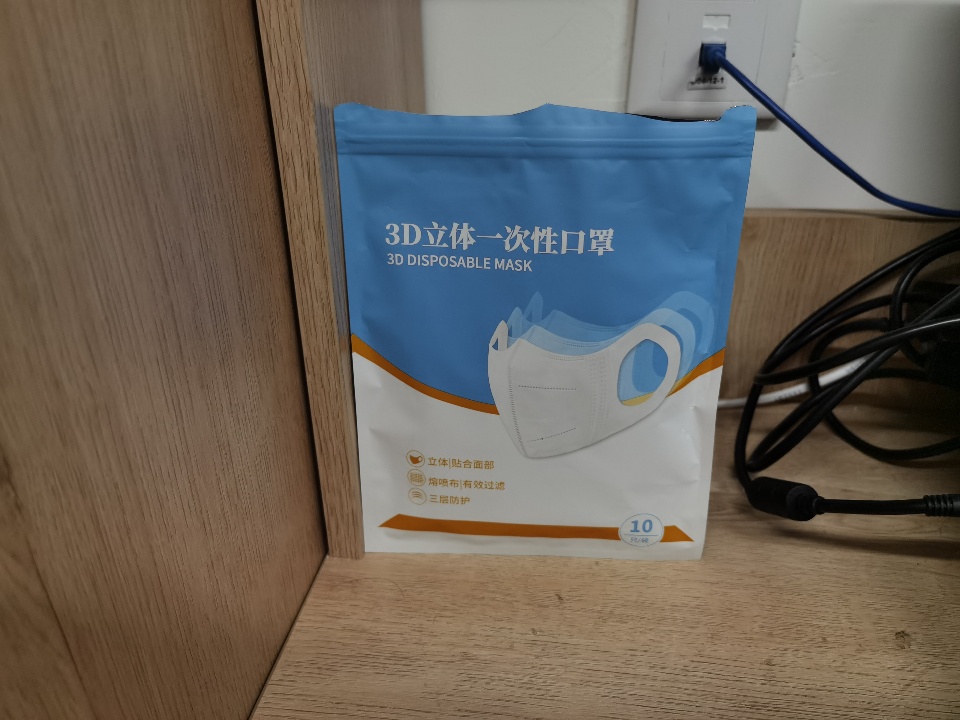

Supplement: S1 File — (ZIP) [file pone.0297984.s001.zip › EVAL/high/2059.jpg]

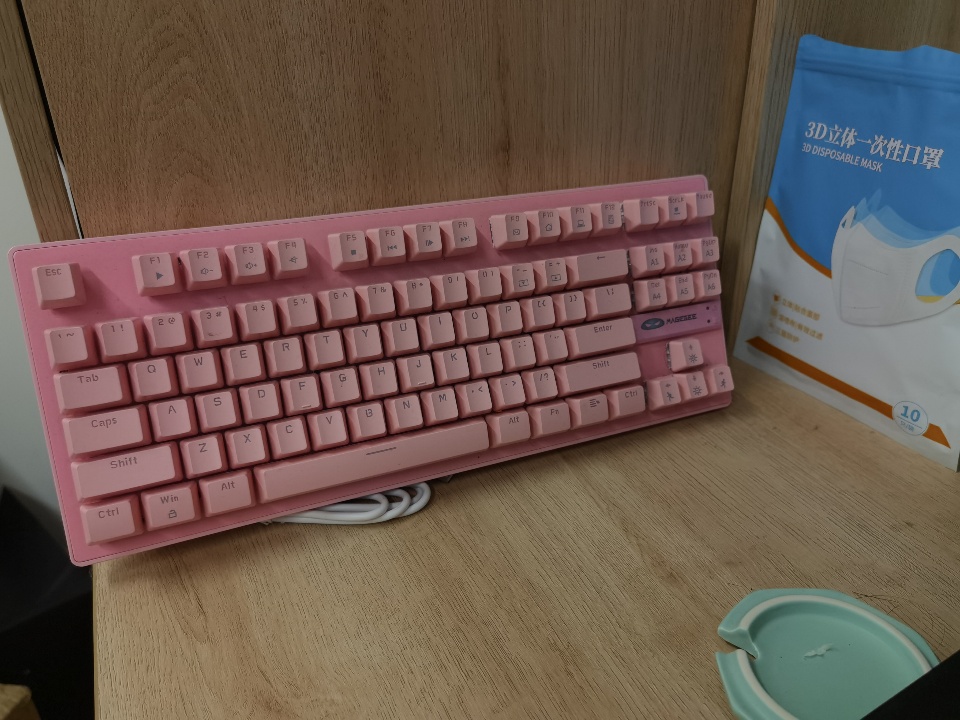

Supplement: S1 File — (ZIP) [file pone.0297984.s001.zip › EVAL/high/2060.jpg]

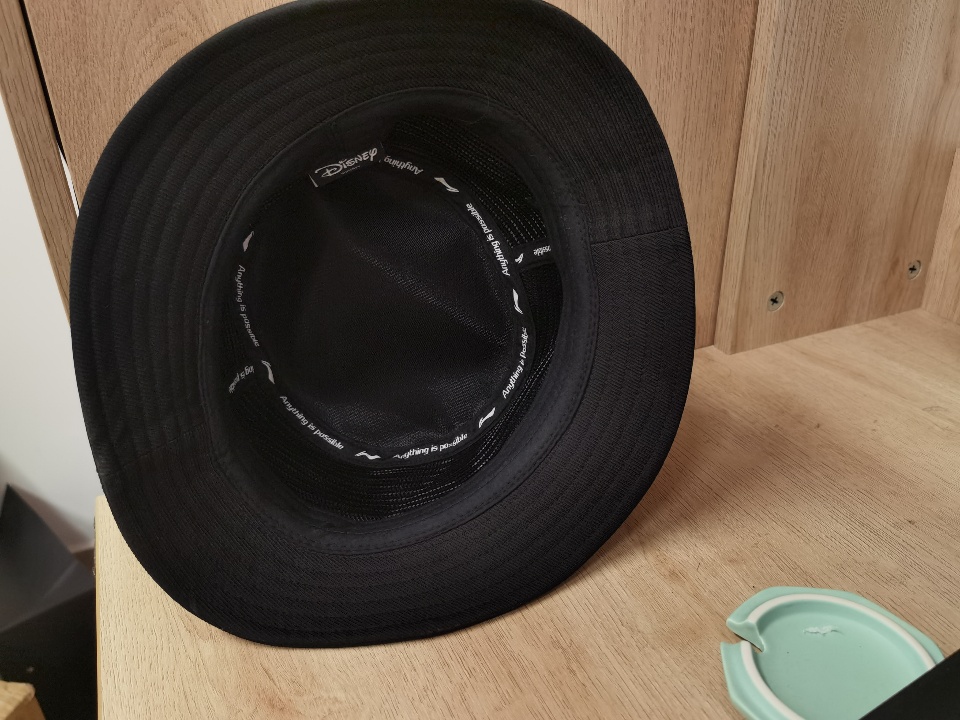

Supplement: S1 File — (ZIP) [file pone.0297984.s001.zip › EVAL/high/2061.jpg]

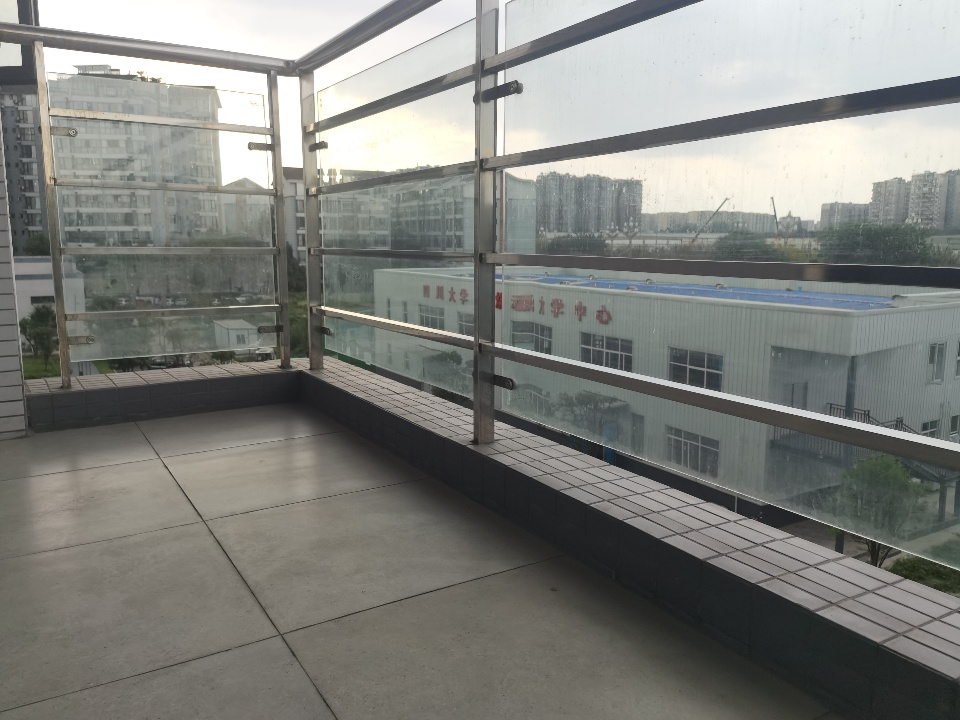

Supplement: S1 File — (ZIP) [file pone.0297984.s001.zip › EVAL/high/2062.jpg]

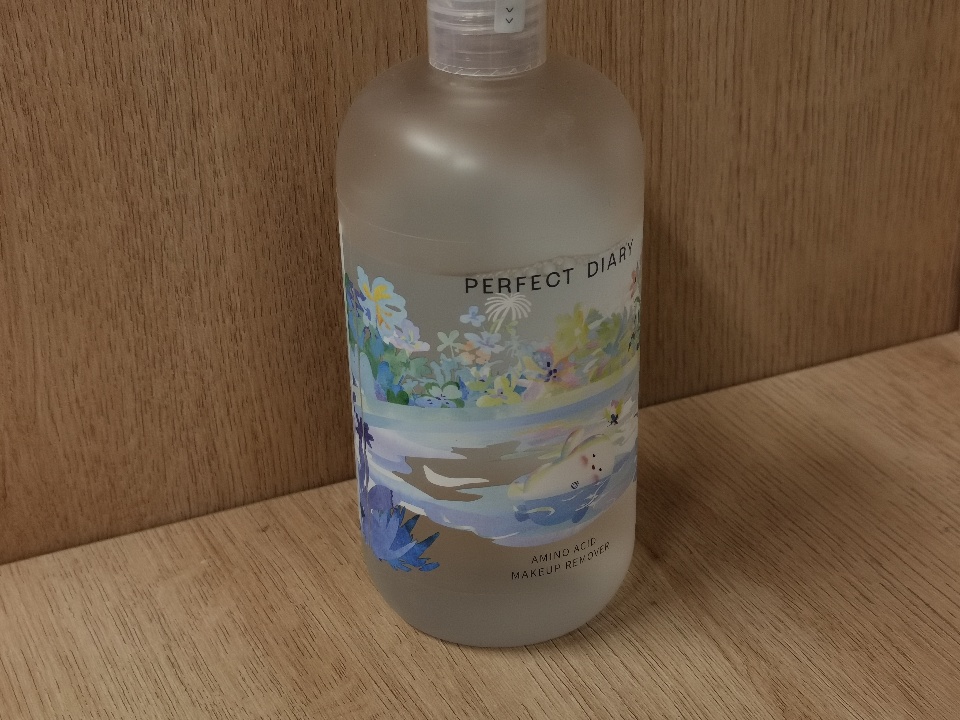

Supplement: S1 File — (ZIP) [file pone.0297984.s001.zip › EVAL/high/2063.jpg]

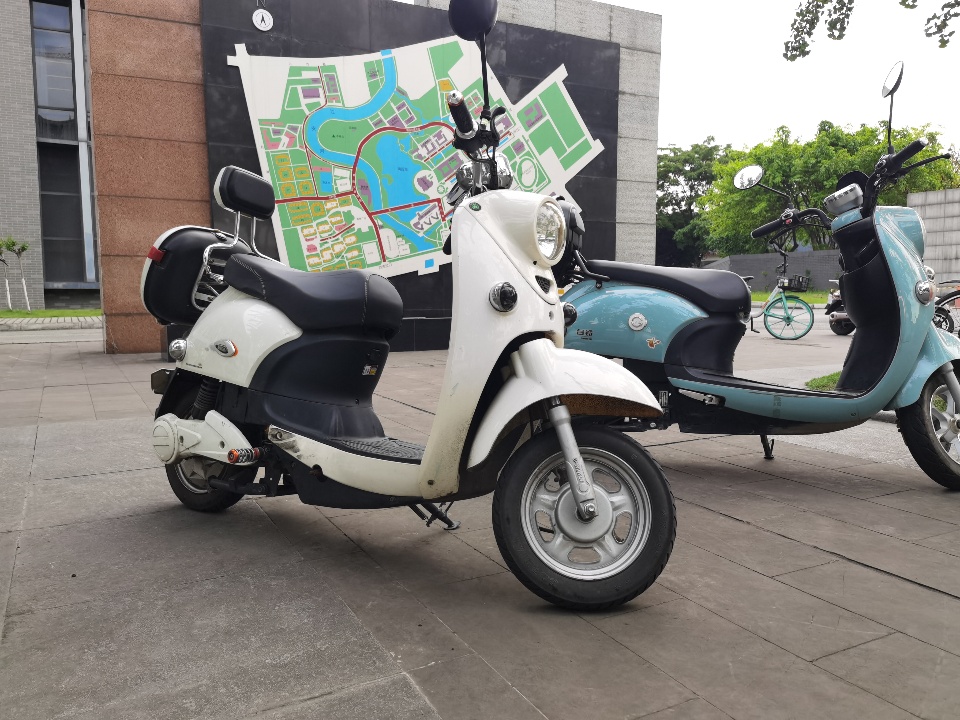

Supplement: S1 File — (ZIP) [file pone.0297984.s001.zip › EVAL/high/2064.jpg]

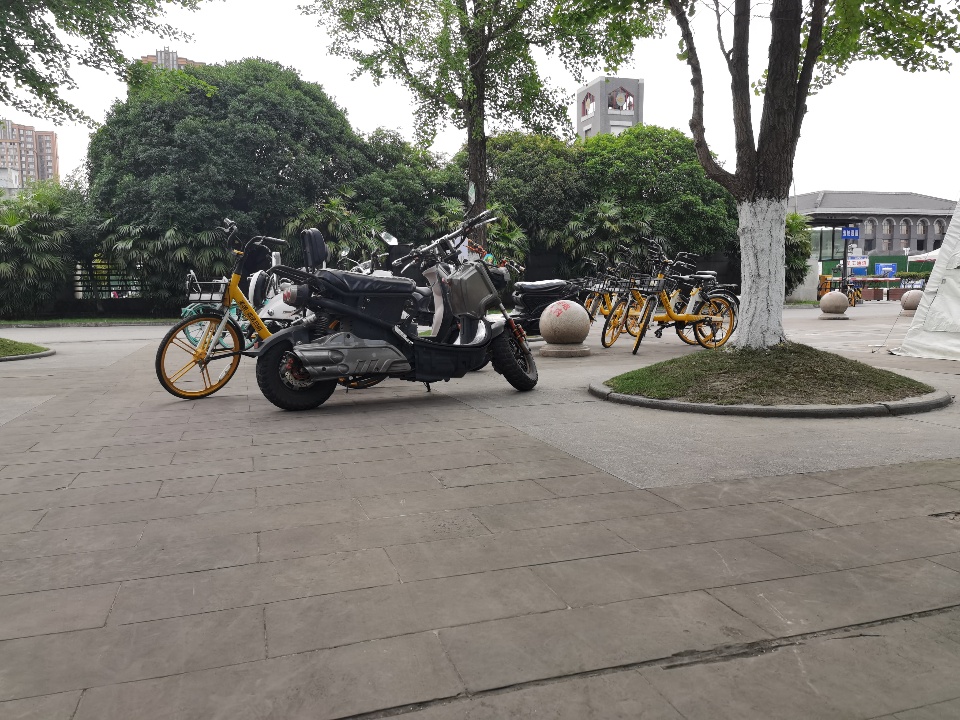

Supplement: S1 File — (ZIP) [file pone.0297984.s001.zip › EVAL/high/2065.jpg]

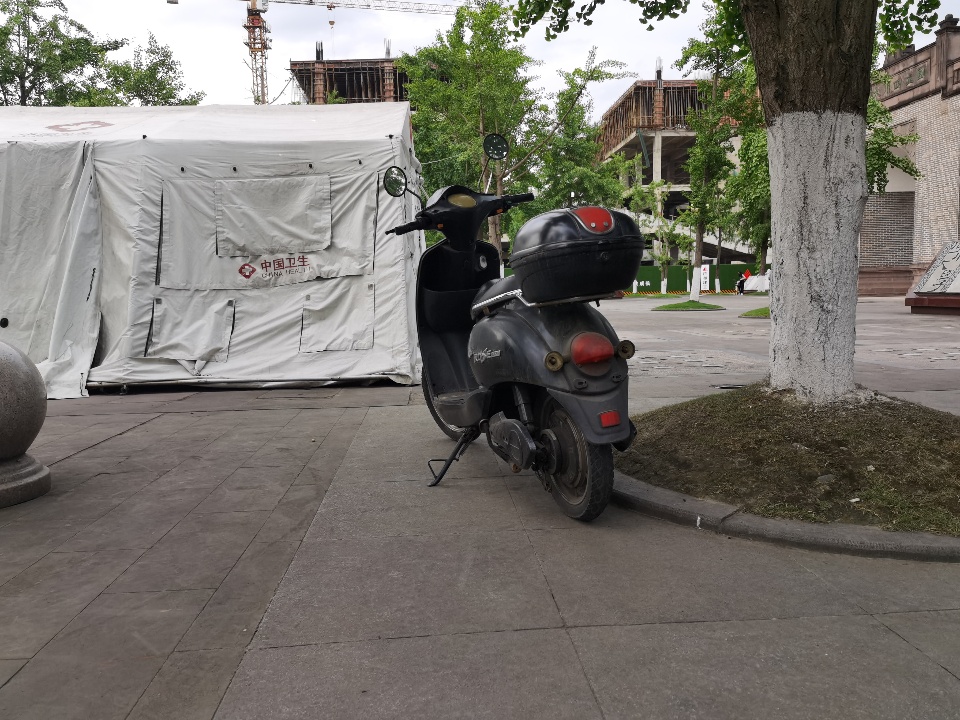

Supplement: S1 File — (ZIP) [file pone.0297984.s001.zip › EVAL/high/2066.jpg]

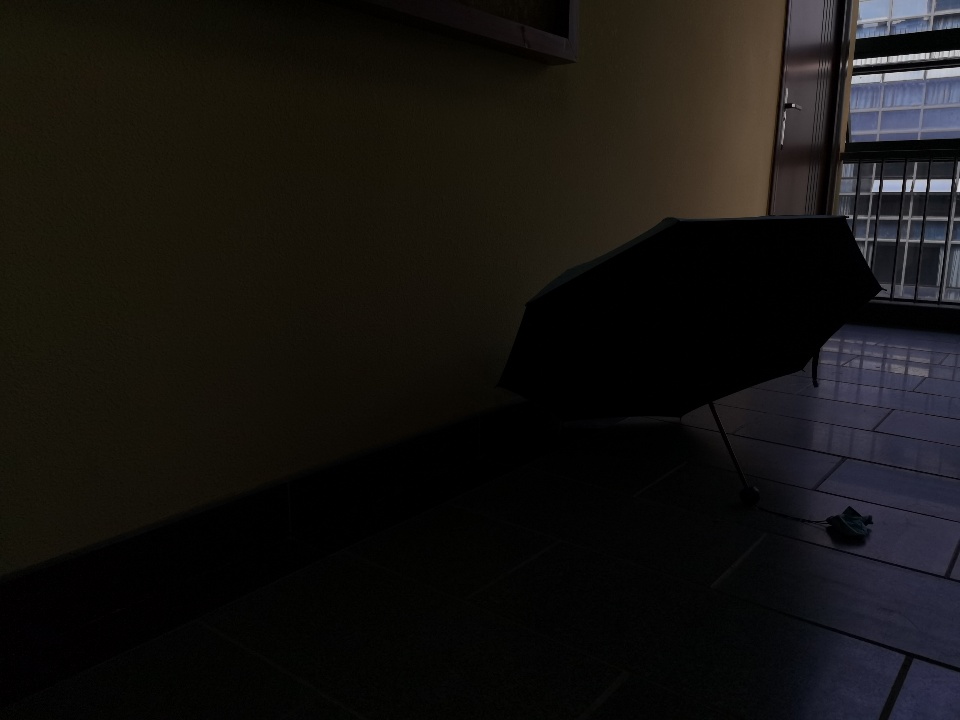

Supplement: S1 File — (ZIP) [file pone.0297984.s001.zip › EVAL/low/2037.jpg]

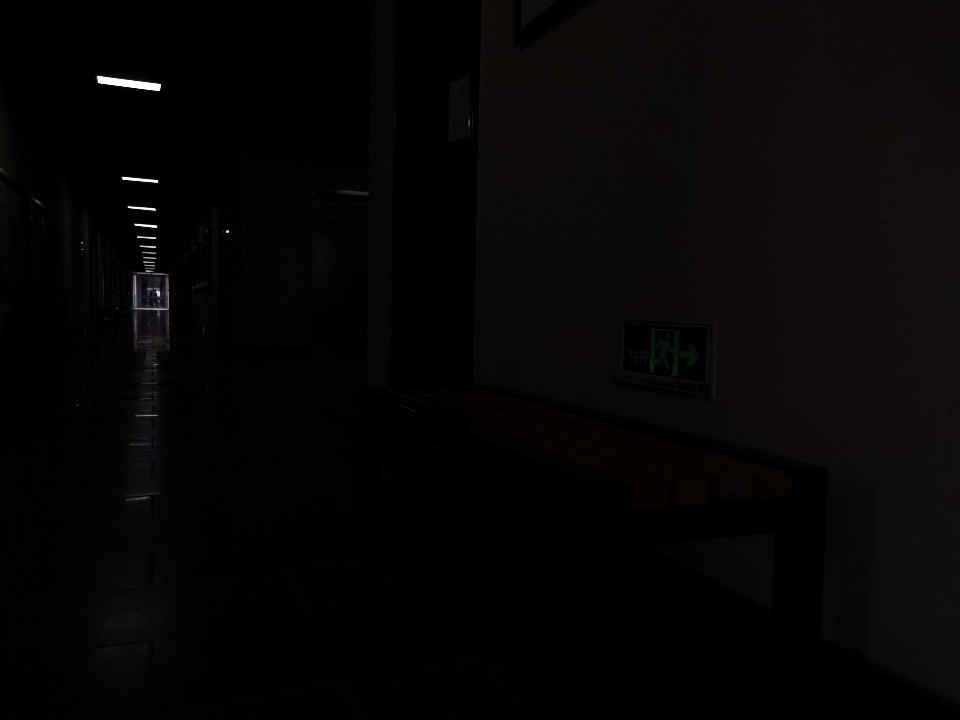

Supplement: S1 File — (ZIP) [file pone.0297984.s001.zip › EVAL/low/2038.jpg]

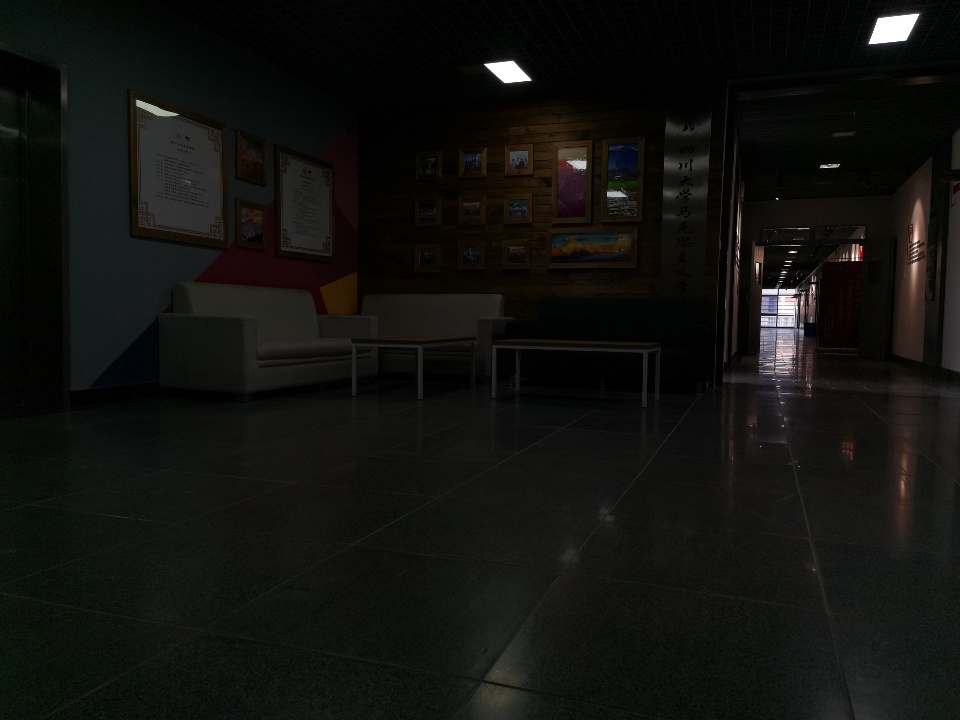

Supplement: S1 File — (ZIP) [file pone.0297984.s001.zip › EVAL/low/2039.jpg]

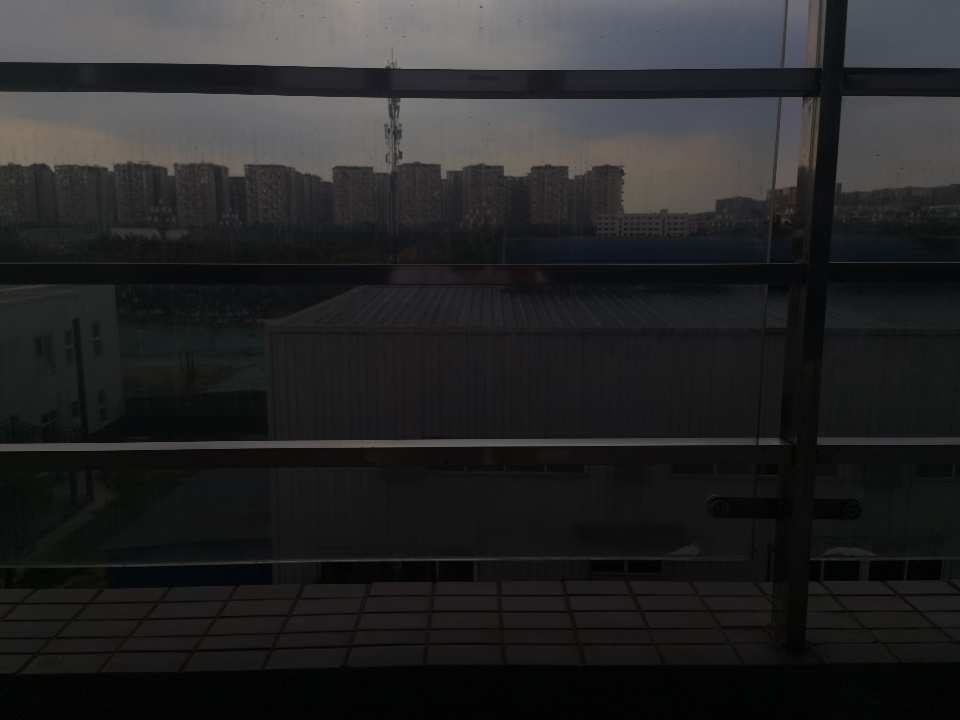

Supplement: S1 File — (ZIP) [file pone.0297984.s001.zip › EVAL/low/2040.jpg]

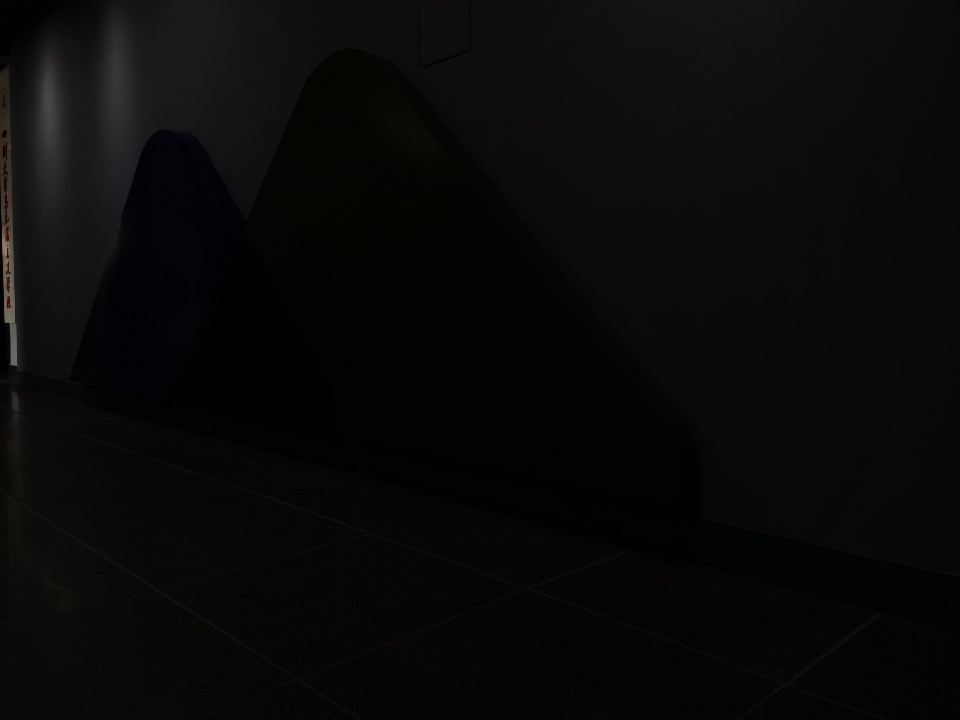

Supplement: S1 File — (ZIP) [file pone.0297984.s001.zip › EVAL/low/2041.jpg]

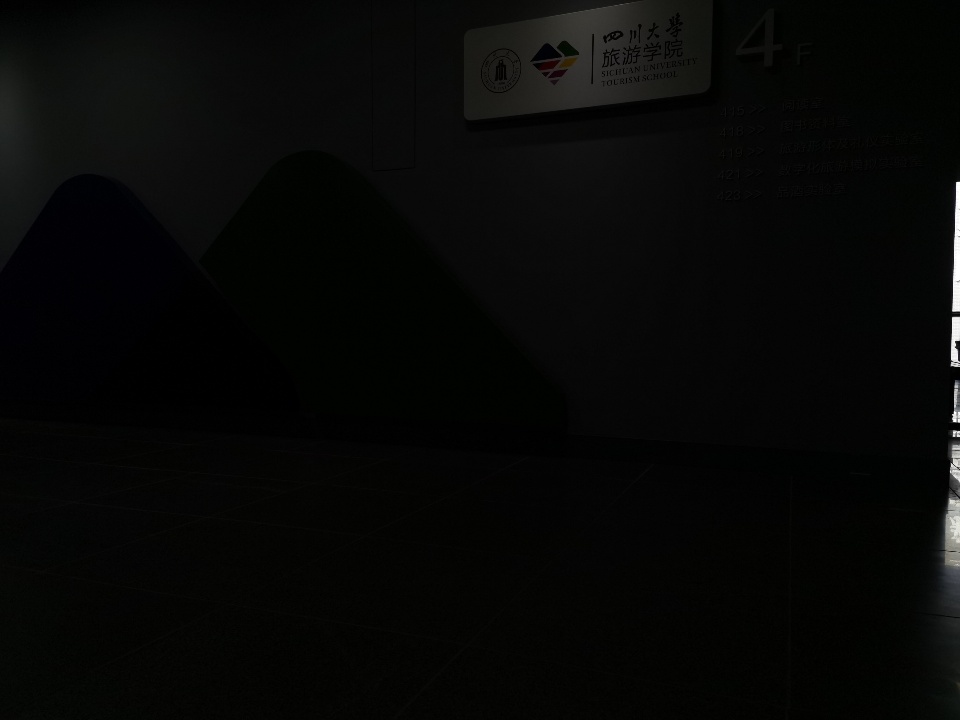

Supplement: S1 File — (ZIP) [file pone.0297984.s001.zip › EVAL/low/2042.jpg]

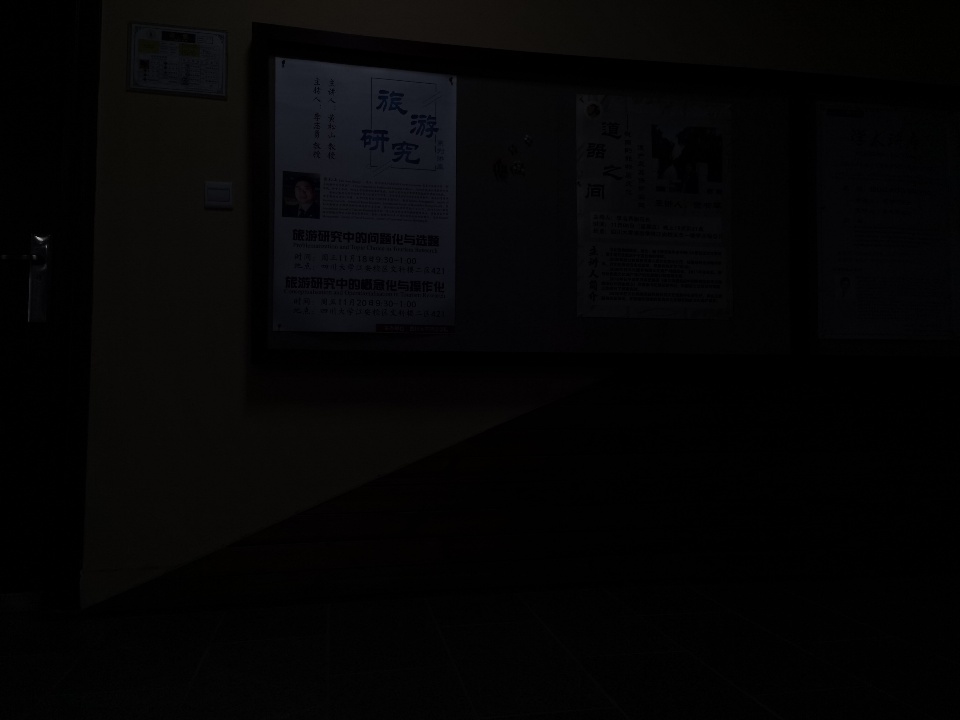

Supplement: S1 File — (ZIP) [file pone.0297984.s001.zip › EVAL/low/2043.jpg]

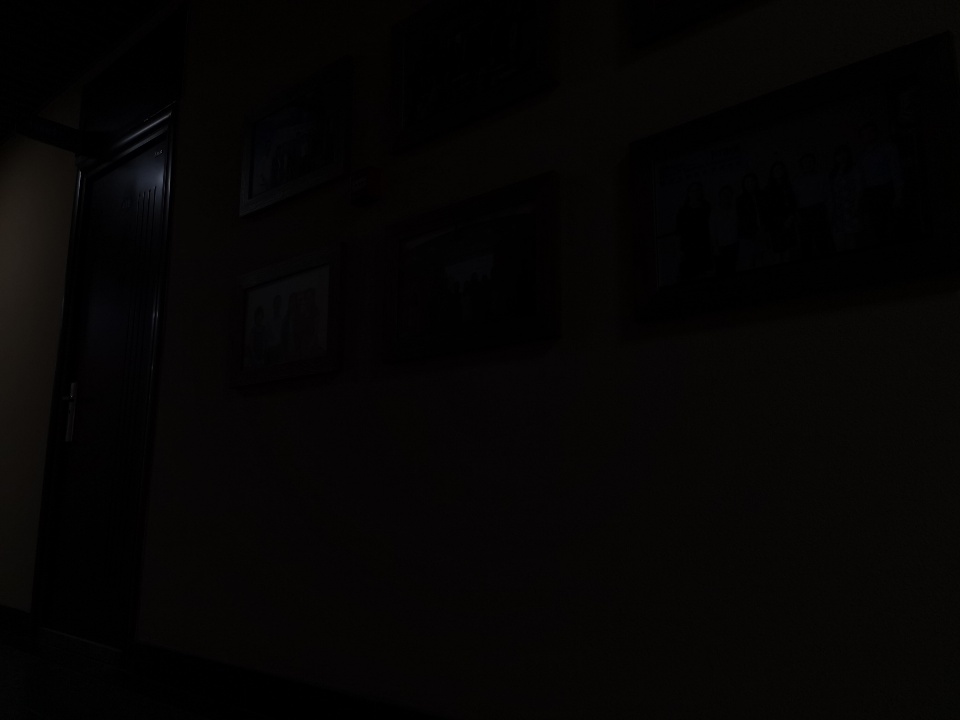

Supplement: S1 File — (ZIP) [file pone.0297984.s001.zip › EVAL/low/2044.jpg]

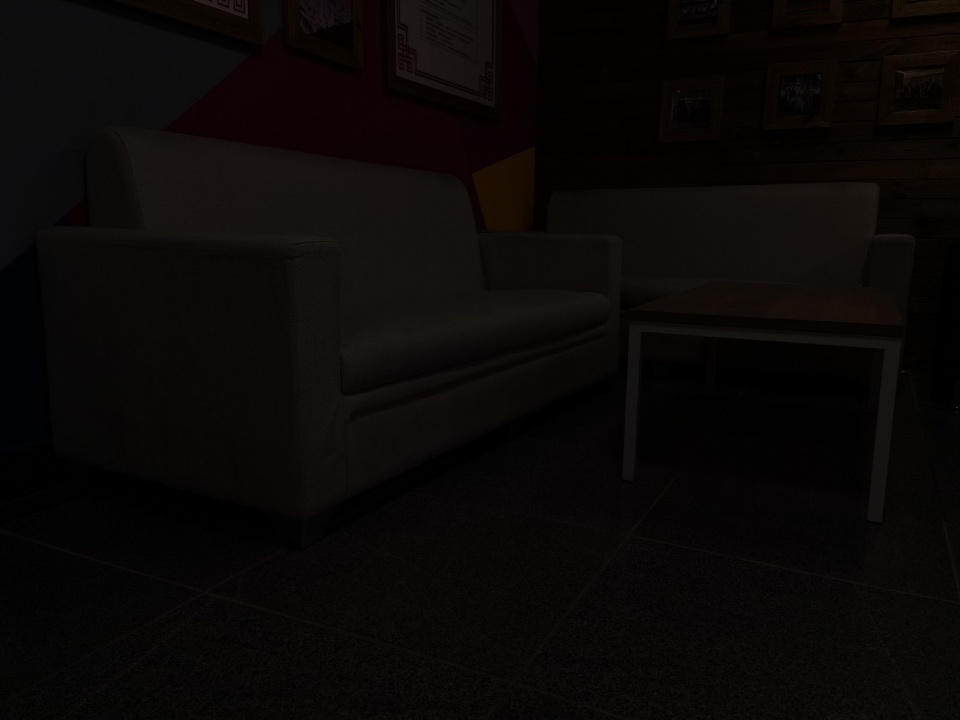

Supplement: S1 File — (ZIP) [file pone.0297984.s001.zip › EVAL/low/2045.jpg]

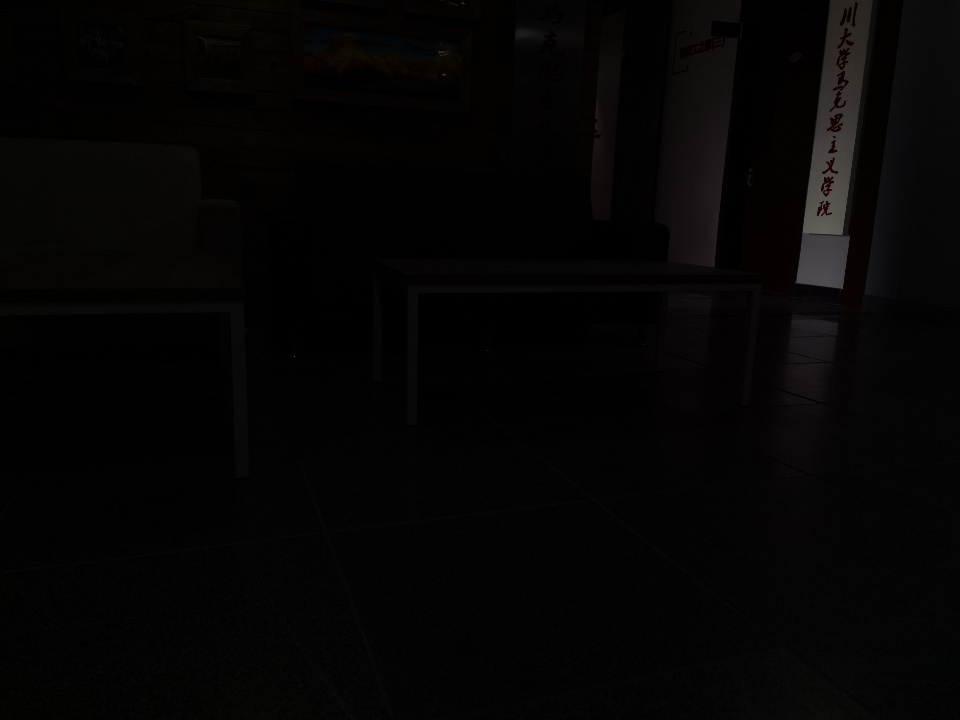

Supplement: S1 File — (ZIP) [file pone.0297984.s001.zip › EVAL/low/2046.jpg]

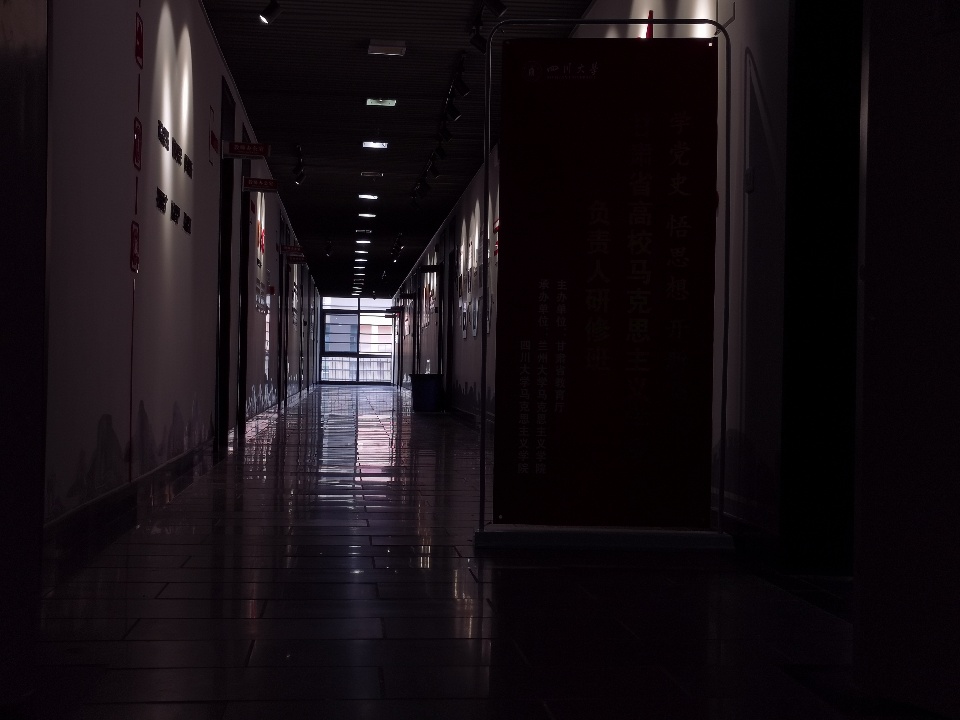

Supplement: S1 File — (ZIP) [file pone.0297984.s001.zip › EVAL/low/2047.jpg]

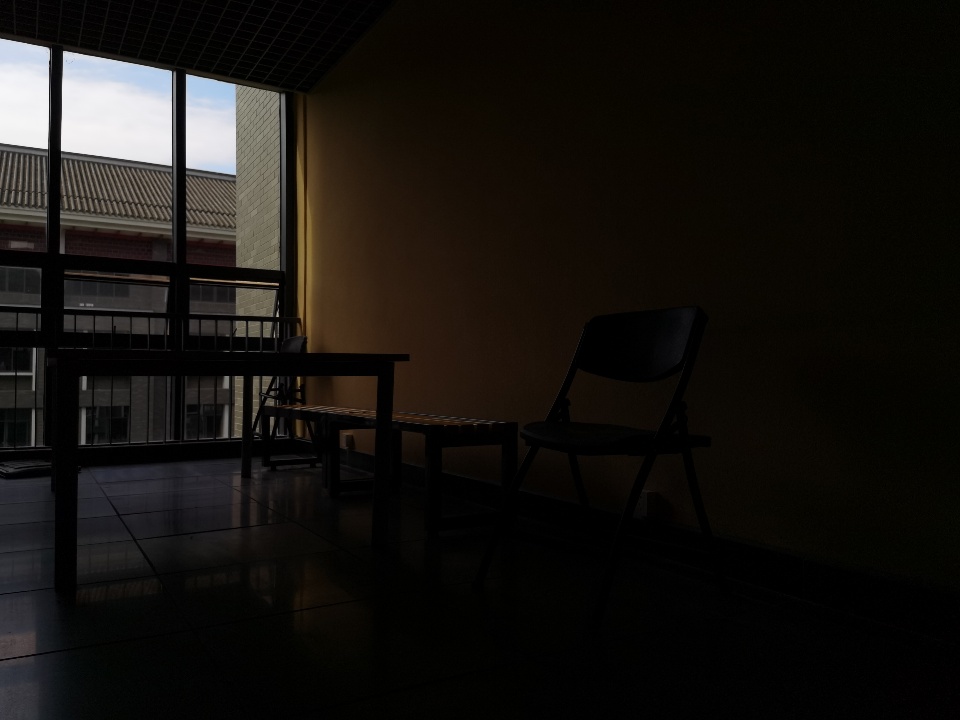

Supplement: S1 File — (ZIP) [file pone.0297984.s001.zip › EVAL/low/2048.jpg]

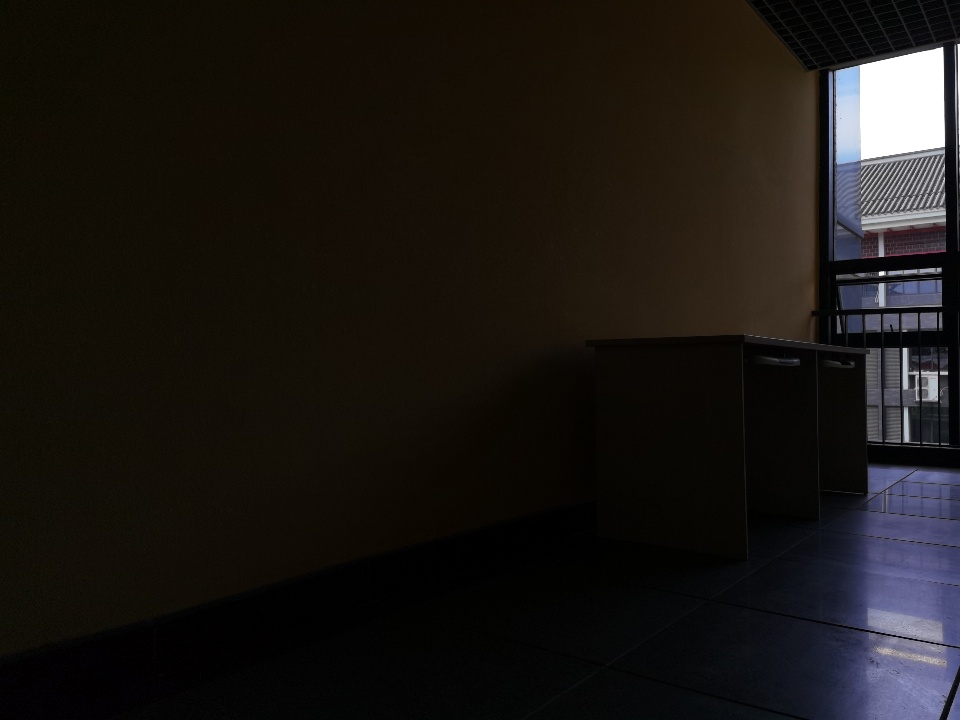

Supplement: S1 File — (ZIP) [file pone.0297984.s001.zip › EVAL/low/2049.jpg]

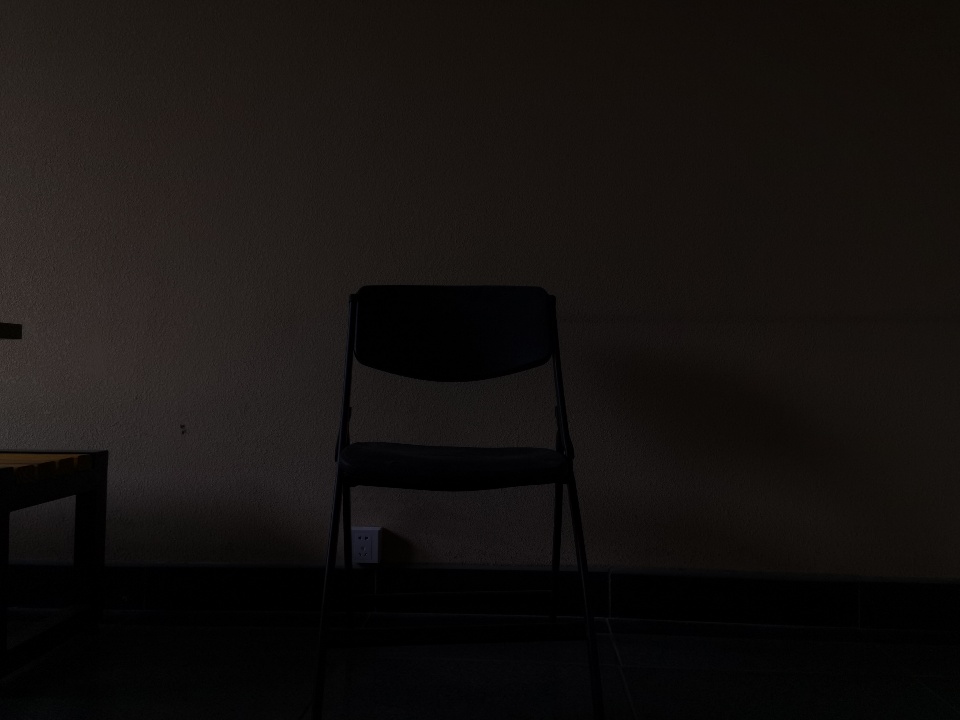

Supplement: S1 File — (ZIP) [file pone.0297984.s001.zip › EVAL/low/2050.jpg]

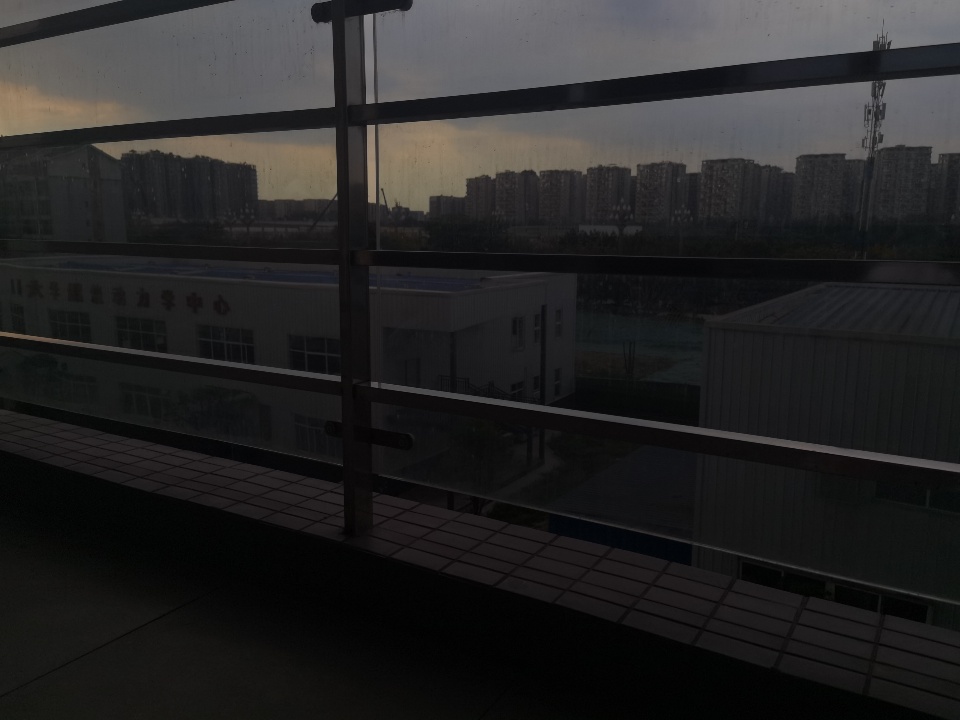

Supplement: S1 File — (ZIP) [file pone.0297984.s001.zip › EVAL/low/2051.jpg]

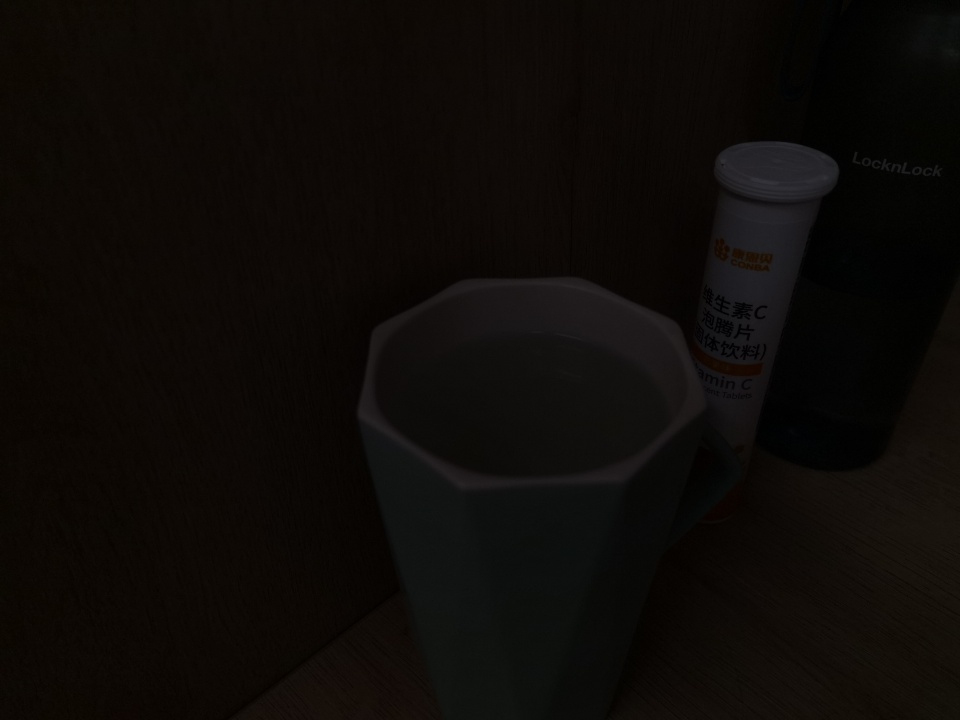

Supplement: S1 File — (ZIP) [file pone.0297984.s001.zip › EVAL/low/2052.jpg]

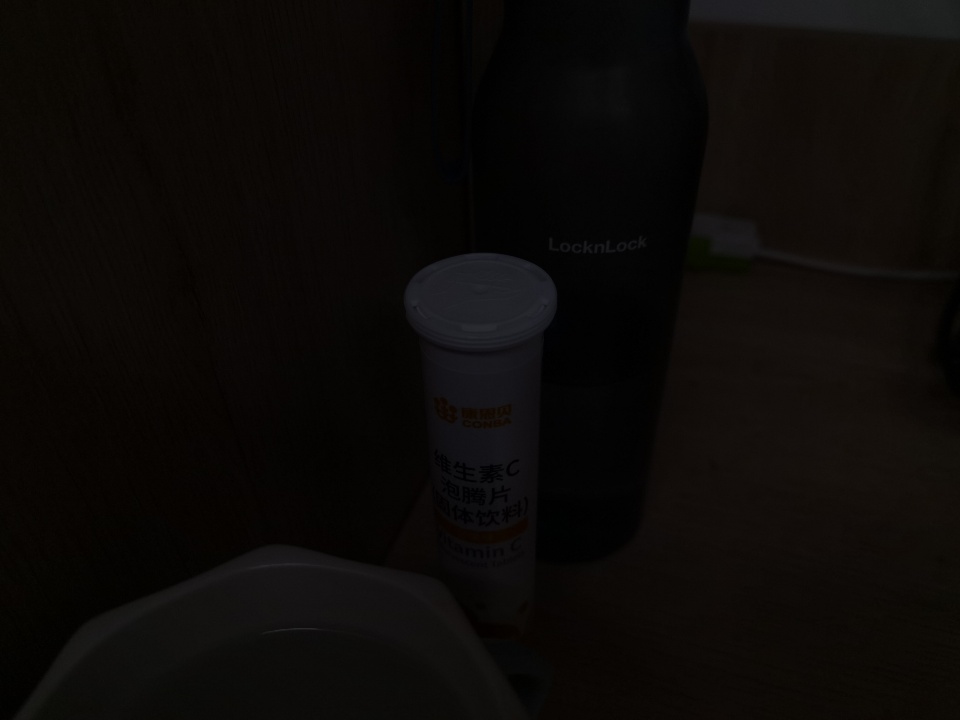

Supplement: S1 File — (ZIP) [file pone.0297984.s001.zip › EVAL/low/2053.jpg]

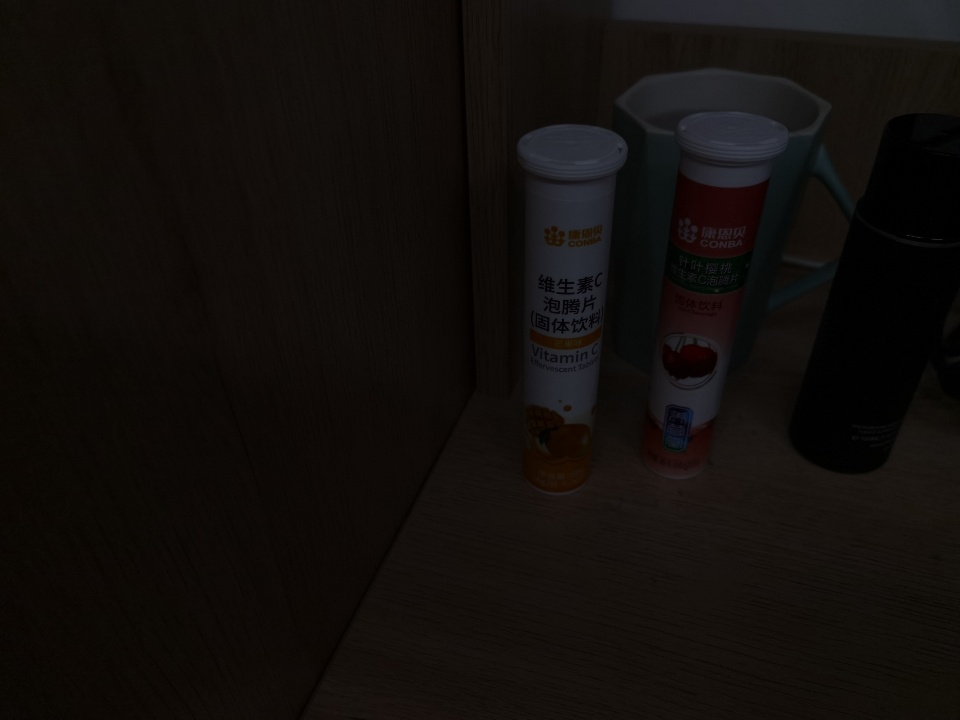

Supplement: S1 File — (ZIP) [file pone.0297984.s001.zip › EVAL/low/2054.jpg]

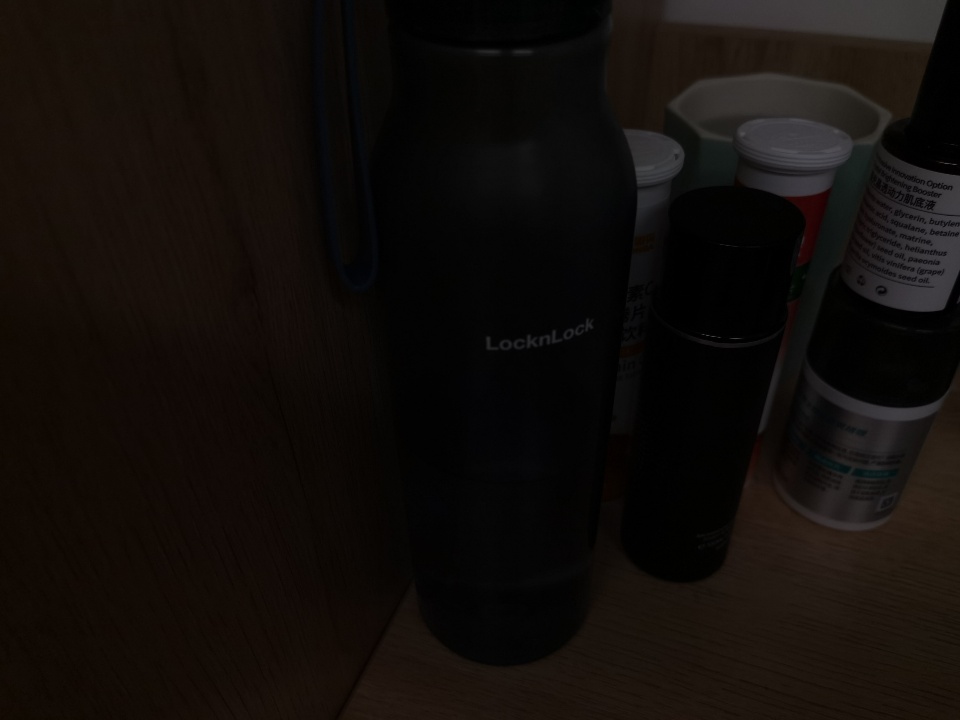

Supplement: S1 File — (ZIP) [file pone.0297984.s001.zip › EVAL/low/2055.jpg]

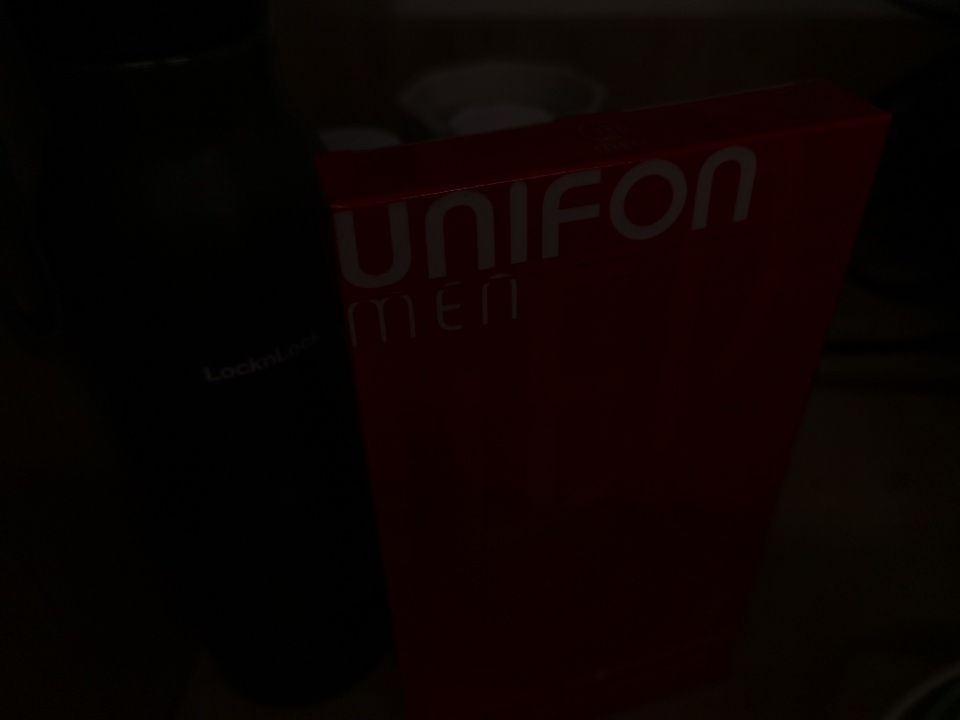

Supplement: S1 File — (ZIP) [file pone.0297984.s001.zip › EVAL/low/2056.jpg]

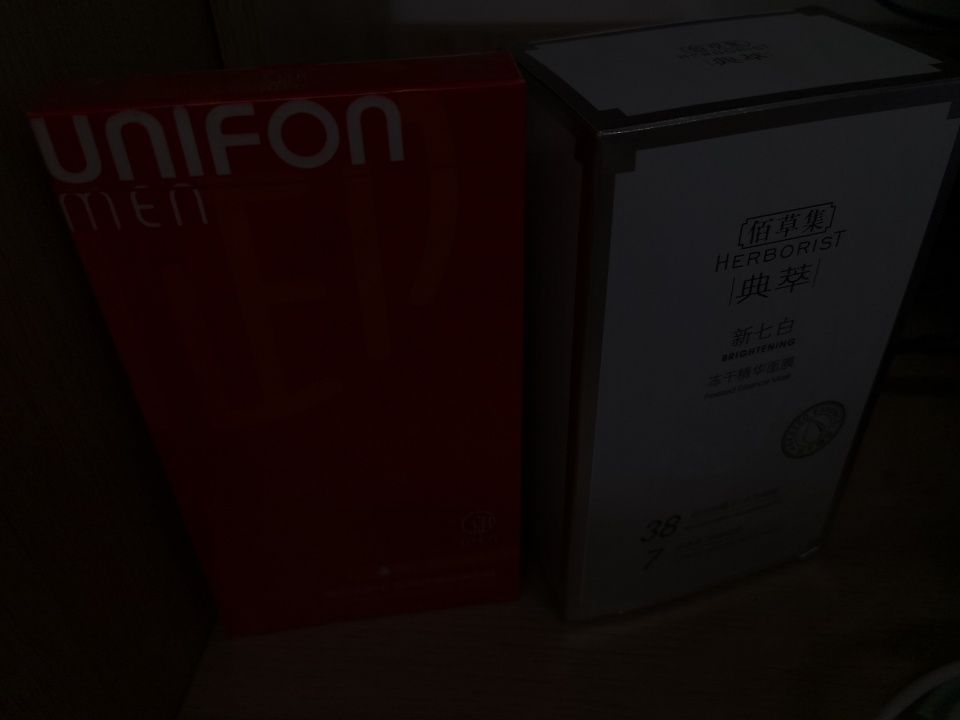

Supplement: S1 File — (ZIP) [file pone.0297984.s001.zip › EVAL/low/2057.jpg]

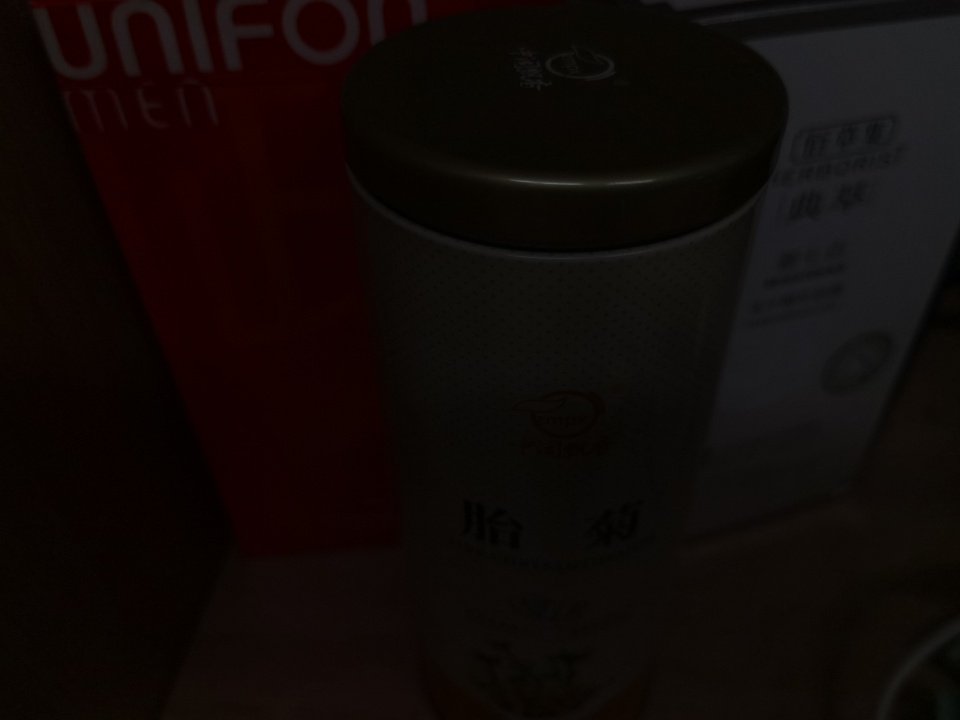

Supplement: S1 File — (ZIP) [file pone.0297984.s001.zip › EVAL/low/2058.jpg]

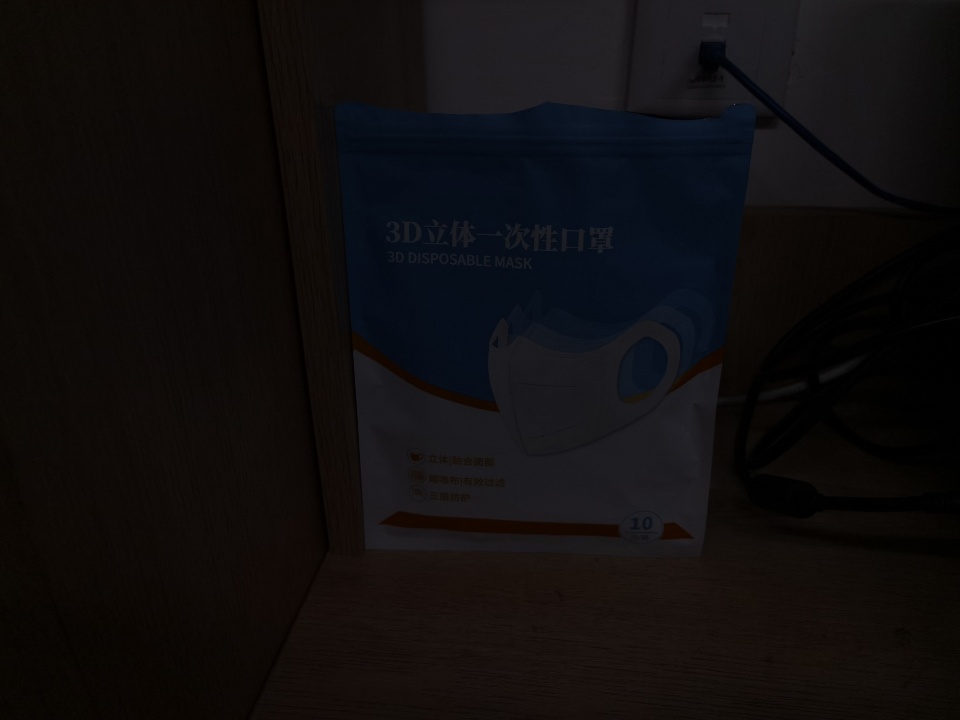

Supplement: S1 File — (ZIP) [file pone.0297984.s001.zip › EVAL/low/2059.jpg]

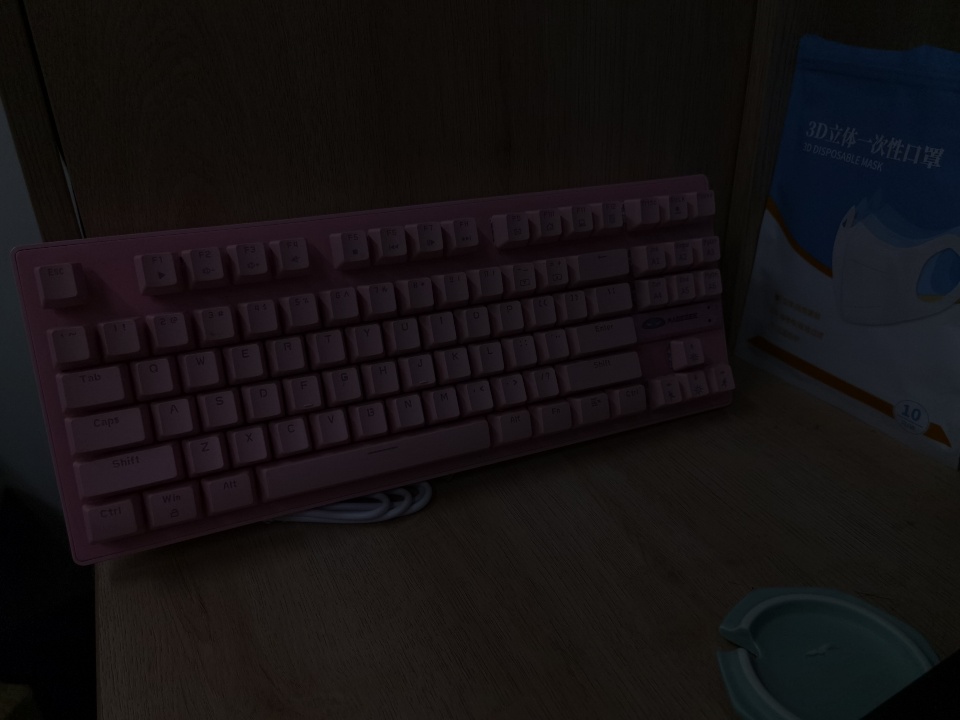

Supplement: S1 File — (ZIP) [file pone.0297984.s001.zip › EVAL/low/2060.jpg]

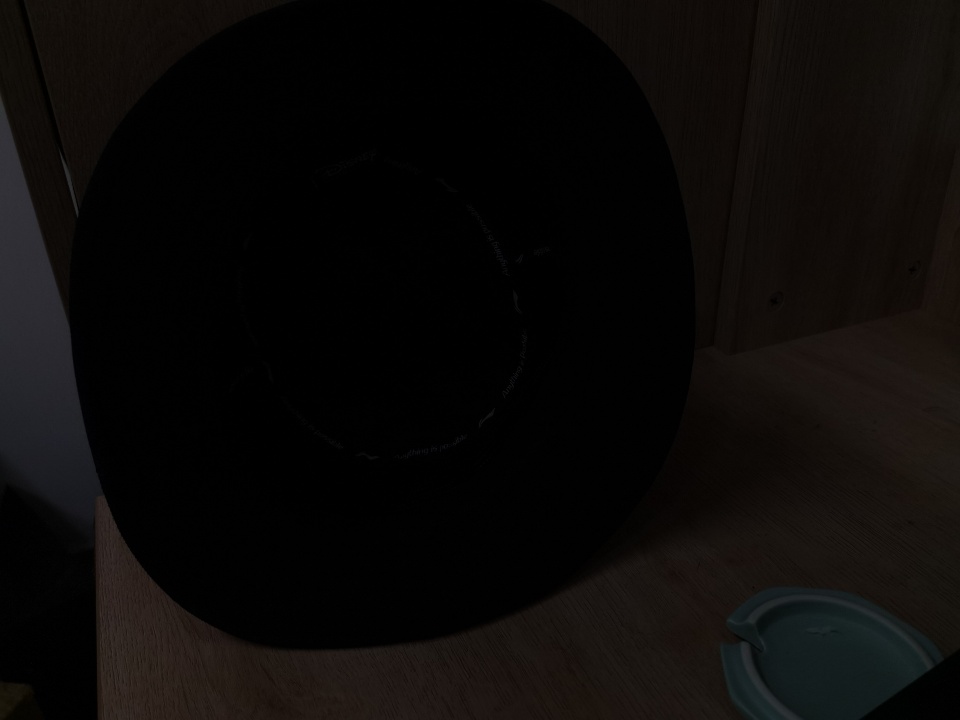

Supplement: S1 File — (ZIP) [file pone.0297984.s001.zip › EVAL/low/2061.jpg]

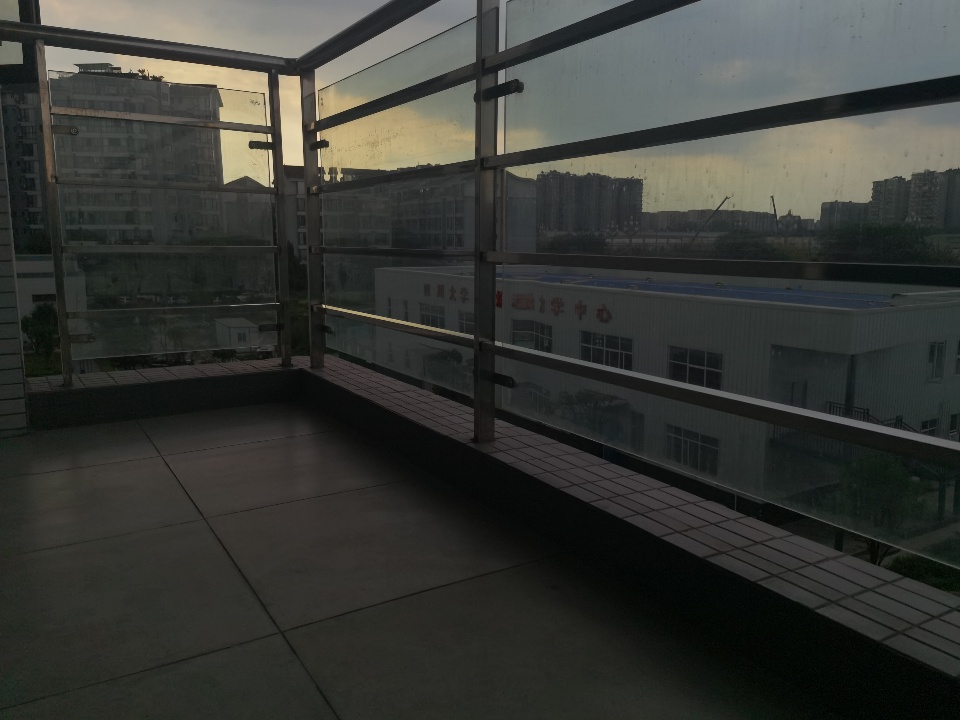

Supplement: S1 File — (ZIP) [file pone.0297984.s001.zip › EVAL/low/2062.jpg]

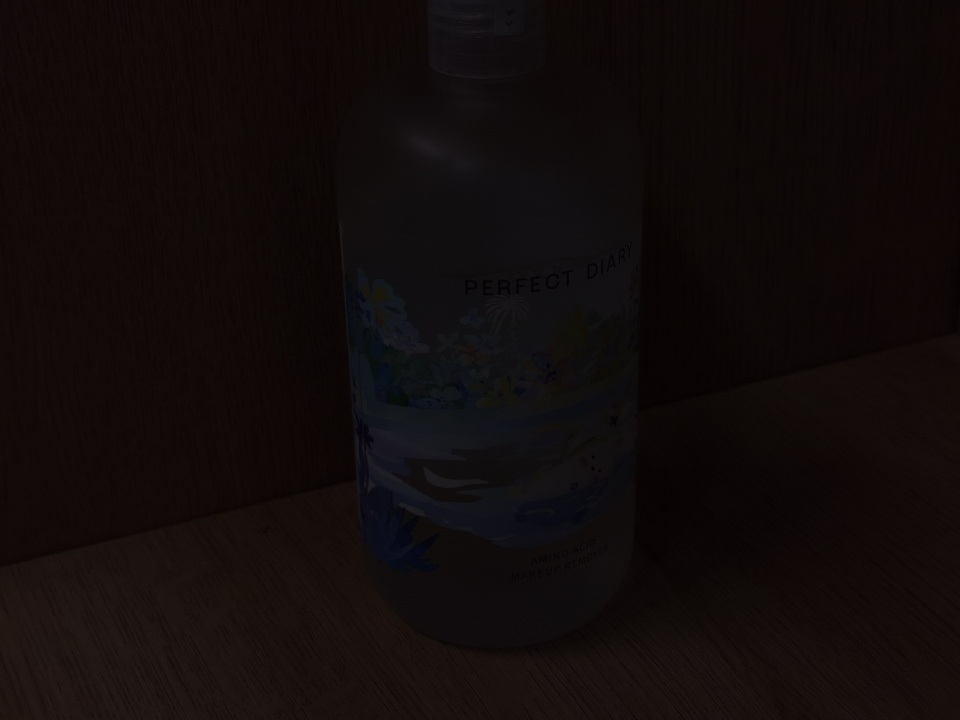

Supplement: S1 File — (ZIP) [file pone.0297984.s001.zip › EVAL/low/2063.jpg]

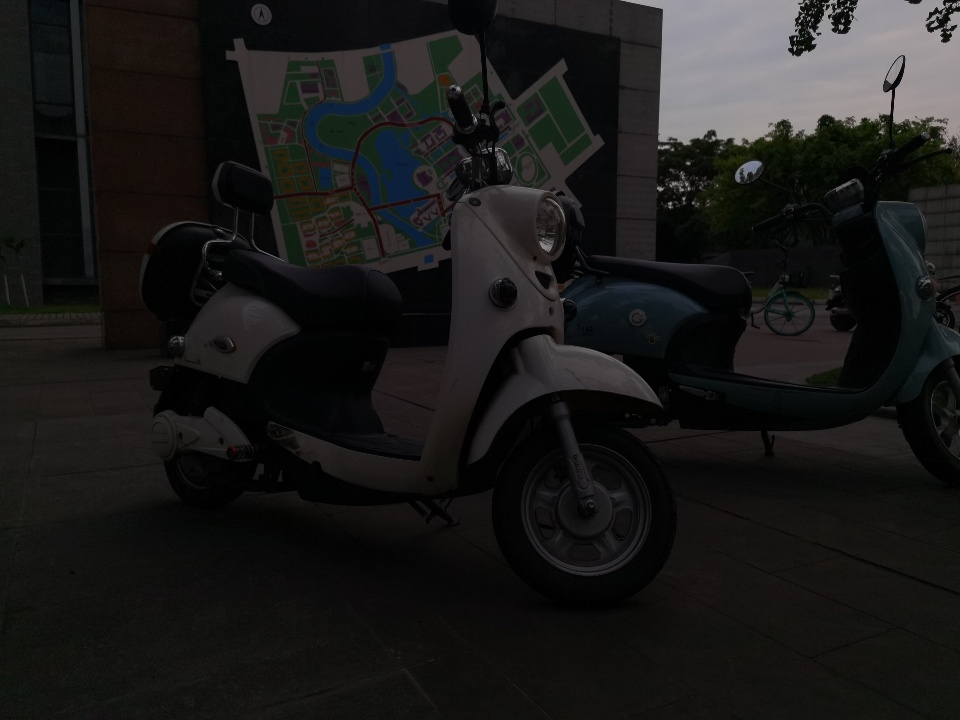

Supplement: S1 File — (ZIP) [file pone.0297984.s001.zip › EVAL/low/2064.jpg]

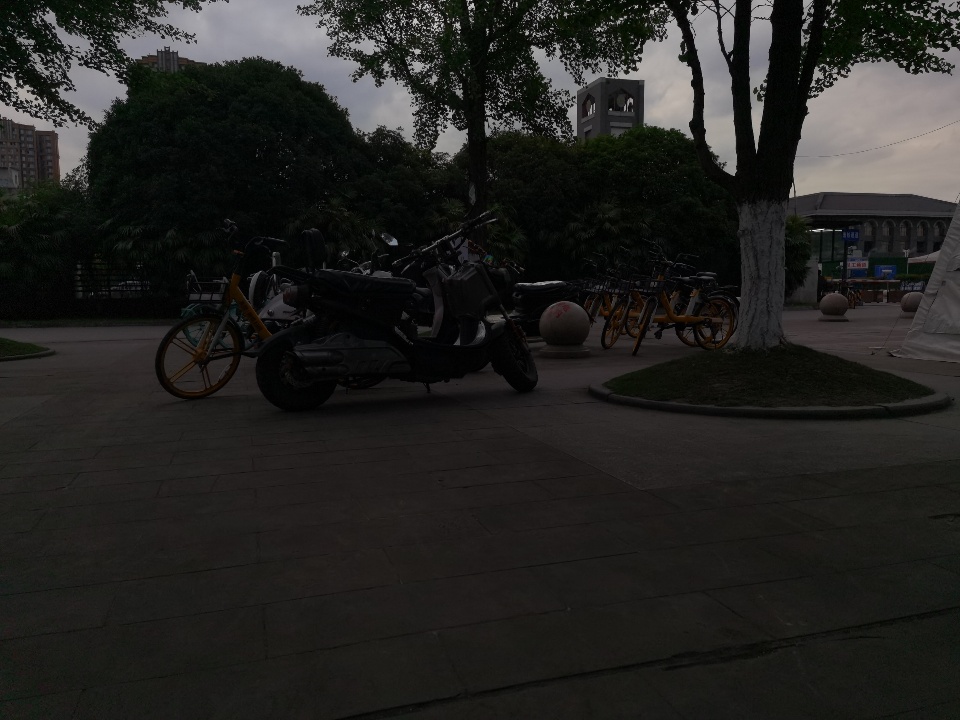

Supplement: S1 File — (ZIP) [file pone.0297984.s001.zip › EVAL/low/2065.jpg]

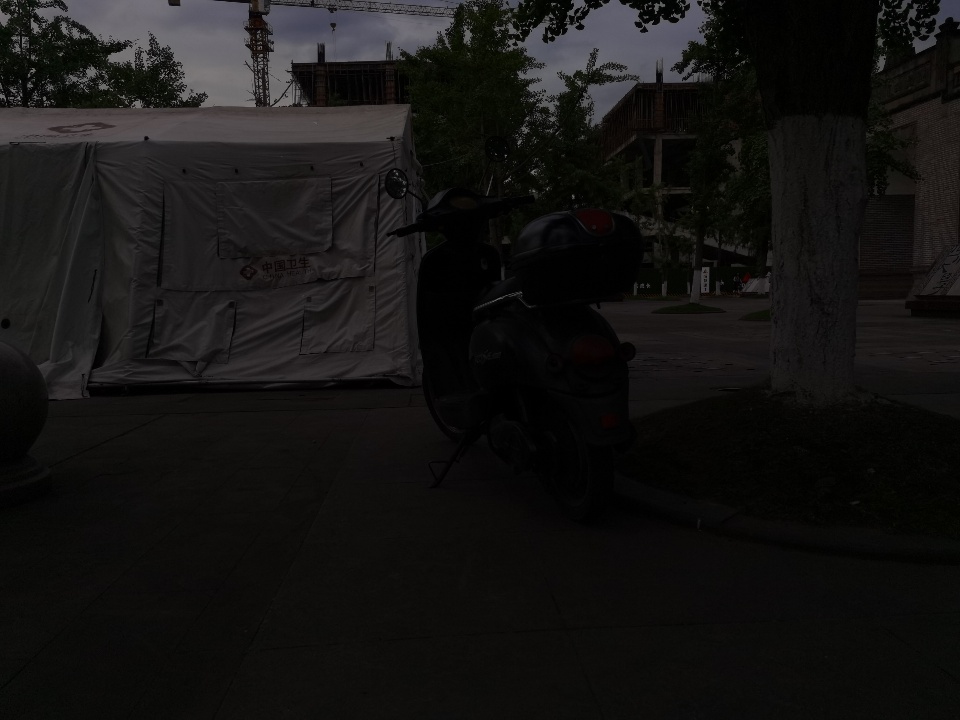

Supplement: S1 File — (ZIP) [file pone.0297984.s001.zip › EVAL/low/2066.jpg]
